# Dissociation and Alterations in Brain Function and Structure: Implications for Borderline Personality Disorder

**DOI:** 10.1007/s11920-017-0757-y

**Published:** 2017-01-30

**Authors:** Annegret Krause-Utz, Rachel Frost, Dorina Winter, Bernet M. Elzinga

**Affiliations:** 1 Institute of Clinical Psychology, Leiden University, Leiden, The Netherlands; 2 Leiden Institute for Brain and Cognition (LIBC), Leiden, The Netherlands; 3 Department of Psychosomatic Medicine and Psychotherapy, Central Institute of Mental Health, Mannheim, Germany; 4 Medical Faculty, University of Heidelberg, Mannheim, Germany

**Keywords:** Dissociation, Trauma, Borderline personality disorder, Posttraumatic stress disorder (PTSD), Depersonalization disorder, Dissociative identity disorder, Neuroimaging, Brain structure and function

## Abstract

Dissociation involves disruptions of usually integrated functions of consciousness, perception, memory, identity, and affect (e.g., depersonalization, derealization, numbing, amnesia, and analgesia). While the precise neurobiological underpinnings of dissociation remain elusive, neuroimaging studies in disorders, characterized by high dissociation (e.g., depersonalization/derealization disorder (DDD), dissociative identity disorder (DID), dissociative subtype of posttraumatic stress disorder (D-PTSD)), have provided valuable insight into brain alterations possibly underlying dissociation. Neuroimaging studies in borderline personality disorder (BPD), investigating links between altered brain function/structure and dissociation, are still relatively rare. In this article, we provide an overview of neurobiological models of dissociation, primarily based on research in DDD, DID, and D-PTSD. Based on this background, we review recent neuroimaging studies on associations between dissociation and altered brain function and structure in BPD. These studies are discussed in the context of earlier findings regarding methodological differences and limitations and concerning possible implications for future research and the clinical setting.

## Introduction

Dissociation is a complex heterogeneous phenomenon. It has been defined as a “disruption of and/or discontinuity in the normal, subjective integration of one or more aspects of psychological functioning, including – but not limited to – memory, identity, consciousness, perception, and motor control” [1, p. 826]. This definition implicates a wide range of psychological symptoms (e.g., depersonalization, derealization, emotional numbing, and memory fragmentations) and somatoform symptoms (e.g., analgesia) [2–4]. Aside from the inability to access normally amenable information and control motor processes (negative symptoms), dissociation includes involuntary intrusions of sensory, affective, and cognitive information into conscious awareness or behavior, e.g., dissociative flashbacks (positive symptoms) [3]. Dissociation can be conceptualized both as a general tendency (trait dissociation) and transient state (state dissociation) and can also be observed in nonclinical populations, albeit at a much lesser extent than in clinical populations [2, 4].

Pathological dissociation is a trans-diagnostic phenomenon, highly prevalent in dissociative disorders and in trauma-related disorders, including depersonalization/derealization disorder (DDD), dissociative identity disorder (DID), posttraumatic stress disorder (PTSD), and borderline personality disorder (BPD) [1, 5]. With respect to PTSD, the most recent version of the *Diagnostic and Statistical Manual of Mental Disorders* (DSM-V) includes a dissociative subtype (dissociative subtype of posttraumatic stress disorder, D-PTSD), characterized by predominately dissociative responses to traumatic reminders and other stressors in the form of depersonalization and/or derealization [5]. In BPD, dissociation is primarily stress-related and appears to have substantial impact on affective–cognitive functioning [6–8].

The precise neural underpinnings of dissociation are still unclear. Yet, neuroimaging research in clinical samples characterized by high dissociation (e.g., DDD, DID, and D-PTSD) have already provided valuable insight into structural and functional networks of brain regions possibly involved in dissociation [9, 10, 11•]. Compared to this relatively large body of literature, neuroimaging studies on dissociation in BPD are still relatively rare.

The present article gives an overview of recent neuroimaging studies in BPD examining associations between state/trait dissociation and altered brain structure and function. Disentangling disorder-specific effects is complicated, as disorders characterized by high dissociation (e.g., BPD, D-PTSD, and dissociative disorders) are highly comorbid and may share etiological factors, such as psychological trauma. Therefore, our present article has two objectives: first, we aim to provide an overview of etiological and neurobiological models of dissociation, primarily based on previous findings in DDD, DID, and D-PTSD. A complete review of this literature is beyond the scope of this article; therefore, for more extensive reviews, see, e.g., [1, 9, 10, 11•]. Our second aim is to discuss recent neuroimaging studies (including measures of state/trait dissociation) in BPD, with respect to key findings related to dissociation, methodological differences and limitations, and possible implications for future research and the clinical setting.

### Etiological Models: Trauma and Dissociation

Psychological trauma, stress such as severe and chronic childhood abuse/neglect, has been critically implicated in the development of dissociation [1, 10, 11•, 12–17], suggesting a complex interaction of (genetic, neurobiological, and cognitive) predispositions/vulnerabilities and stressful life events [18–20].

Dissociation during traumatic events (also referred to as peritraumatic dissociation [18]) can be considered an adaptive defense mechanism to cope with overwhelming threat that cannot be prevented or escaped [3, 11•]. States of subjective detachment (e.g., depersonalization, derealization, and numbing) may help to create an inner distance to the overwhelming experience by dampening unbearable emotions and reducing conscious awareness of the event. The traumatic situation may be perceived as an unreal film-like scene that is not happening to oneself but observed from a wider distance. Somatoform symptoms such as analgesia and out of body experiences (e.g., the sense of floating above one’s body) may reduce awareness of physical injury [16].

While direct translations between animal and human studies are difficult [21], some models have conceptualized peritraumatic dissociation analogous to the freezing response observed in animals (see, e.g., [16]). The proximity of threat may at first elicit an orienting response, preparing the organism for an active defense mechanism (fight or flight reaction [22]), associated with increased sympathetic nervous system activation (e.g., in heart rate, blood pressure, and release of stress hormones). In situations that cannot be controlled or escaped, the threatened organism may more likely engage in a passive defense mode, accompanied by tonic immobility, increased parasympathic activity, and a “shut-down” of the arousal system [14, 16, 21, 23]. Passive reactions (i.e, tonic immobility) in the face of unescapable threat may enhance survival when the chance of escaping or winning a fight is low or impossible, e.g., by reducing the risk of being detected [23, 24]. As pointed out before, however, translations from animal to human research are complicated by conceptual and methodological differences (see, e.g., [21]).

There is evidence that peritraumatic dissociation increases the risk of subsequent PTSD [18, 25–30]. Although the precise mechanisms remain elusive [18, 28], disturbed information processing, most prominently memory alterations, may play an important role in this relationship [31–34]. Dissociation is thought to interfere with a coherent encoding of salient events [35–37], leading to a fragmentation (compartmentalization) of memory: sensory, affective, and cognitive aspects of the traumatic event are encoded and stored as separate elements, which may later reoccur as implicit intrusive flashback memories, accompanied by strong sensory impressions as if the traumatic event was happening again in the present [29, 38–42].

Moreover, individuals who are highly vulnerable to experience peritraumatic dissociation are more likely to respond in a similar way to traumatic reminders later on in life [43, 44•, 45]. Dissociation can also develop in the aftermath of trauma and generalize across situations, i.e., individuals who learned to dissociate in response to traumatic/stressful situations may be more likely to do so in the presence of even relatively “minor” stressors [10]. Such trauma-related states of consciousness may comprise distortions in time (e.g., flashback memories), thought (e.g., voice hearing in second-person perspective), body (e.g., depersonalization and out of body experiences), and emotional numbing [43, 44•]. Thereby, dissociation can become maladaptive [10] and possibly interfere with treatment [46–50]. Furthermore, it is assumed that dissociation has an impact on brain function, as discussed in more detail below.

#### Models on Brain Structure and Function Associated With Dissociation

Up to now, the precise neural/neurobiological underpinnings of dissociation remain elusive. Yet, a growing number of neuroimaging studies in DDD, DID, and D-PTSD have implicated dissociative symptoms in altered brain structure and function.

Over the past decades, neuroimaging has become one of the most important tools in clinical neurobiology. Techniques such as magnetic resonance imaging (MRI), MR spectroscopy (MRS), positron emission tomography (PET), and diffusion tensor imaging (DTI) are used to study abnormalities in the brain. By detecting changes in blood-oxygen-level-dependent (BOLD) signal (hemodynamic response), functional MRI (fMRI) provides a measure of brain activity and coactivity (functional connectivity) during experimental tasks or resting state, i.e., in the absence of experimental stimulation. PET (e.g., in combination with pharmacologic challenge) can be used to detect changes in glucose metabolism and neurotransmitter systems. MRS assesses concentrations of neurochemical metabolites like glutamate, N-acetylaspartate (NAA), lactate, or choline in the brain. Structural MRI and DTI measure anatomical abnormalities, e.g., in gray or white matter volume.

Several neuroimaging studies have related their findings to higher scores on psychometric scales like the dissociative experiences scale (DES), measuring trait dissociation with the subscales depersonalization/derealization, amnesia, and absorption [51], or the dissociation stress scale (DSS), a measure of state dissociation, including items on psychological and somatic dissociation and one item on aversive inner tension [52–54]. As an attempt to mimic dissociative experiences in everyday-life situations, some studies used script-driven imagery to experimentally induce dissociation [55–59, 60•]: a narrative of an autobiographical situation involving dissociative experiences (“dissociation script,” as compared to an emotionally neutral script) is created together with each participant and presented in an experimental setting (e.g., during fMRI). Participants are instructed to recall the specific situation as vividly as possible. Other studies used pharmacological challenge (e.g., NMDA antagonists and cannabinoids) to induce dissociative symptoms (see [61]). In the following section, neurobiological models of dissociation, primarily based on research in DDD, DID, and D-PTSD, are discussed.

#### Cortico-Limbic Disconnection Model and Neuroimaging Research in Depersonalization Disorder

Already in 1998, Sierra and Berrios proposed that symptoms of depersonalization may be associated with a “disconnection” of a cortico-limbic brain system, involving the amygdala, anterior cingulate cortex (ACC), and prefrontal structures. In this model, depersonalization is more broadly conceptualized as a state of subjective detachment, involving emotional numbing, emptiness of thoughts, analgesia, and hypervigilance [62]. It is assumed that these symptoms are associated with increased activity in the medial prefrontal cortex (mPFC), dorsolateral prefrontal cortex (dlPFC), and ACC [63], brain areas implicated in attention, cognitive control, and arousal modulation. Increased recruitment of the PFC may (both directly and indirectly via the ACC) lead to dampened activity in the amygdala and a marked attenuation of automatic responses, comparable to “shutting down the affective system” [62, 64–66]. The amygdala is fundamentally involved in salience detection and emotion processing such as the initiation of stress and fear responses [63, 67–69]. States of detachment (e.g., numbing) may thus be associated with reduced reactivity in this area [70].

Using fMRI, Phillips and colleagues (2001) investigated brain activity during the presentation of aversive versus neutral images in patients with chronic depersonalization disorder, patients with obsessive-compulsive disorder (OCD), and healthy controls (HC) [69]. In response to aversive images, depersonalization disorder patients reported less arousal and showed diminished activity in the occipito-temporal cortex, ACC, and insula compared to OCD and HCs [69]. The insula plays an important role in attention modulation, encoding of negative emotions, interoceptive awareness, and pain perception [71–75]. Diminished activity in this area may therefore reflect reduced interoceptive/emotional awareness [69, 76•]—an assumption that is supported by a more recent study in chronic depersonalization patients [77•]. In this study by Lemche and colleagues, altered anterior insula and dorsal ACC reactivity to sad emotional expressions was associated with traits of alexithymia, i.e., difficulties in identifying and describing feelings. In another study of this group [78•], a stronger coupling of the dorsomedial PFC (Brodmann area (BA) 9) and posterior cingulate cortex (PCC; BA31) was found in depersonalization disorder patients [78•]. The PCC is a critical node of the default mode network (DMN), a brain network that has been implicated in “inward-directed” (self-referential) processes, such as episodic memory encoding/retrieval, self-monitoring, daydreaming, planning, rumination, and pain processing [79–81]. Further evidence for altered self-referential processing in depersonalization disorder patients stems from an fMRI study in which DDD patients were exposed to either their own photographs or a stranger’s face [82•]. While viewing their own photographs, patients showed stronger activity in areas implicated in self-referential processing, e.g., mPFC, which was positively correlated with depersonalization severity [82•].

Brain function in depersonalization disorder may also be altered in the absence of experimental stimulation: in a PET study by Simeon and colleagues (2000), patients with chronic depersonalization disorder demonstrated significantly lower metabolic activity in the right middle/superior temporal gyrus (BA21/22) and higher metabolism in the parietal and occipital areas (BA7, 39, and 19)—metabolic activity in area 7B was positively correlated with clinical depersonalization scores [83]. Altered glucose metabolism in tempo-parietal regions may play a role in “feeling unreal” [83], e.g., altered consciousness, sensory integration, body schema, and memory, as suggested by observations in patients with temporal lobe epilepsy [84] and research on the role of the temporal lobe in memory processing [32].

In sum, there is evidence for altered activity in brain regions associated with emotional and self-referential processing in patients with chronic depersonalization disorder.

#### Models on Emotion Modulation and Research in the Dissociative Subtype of PTSD (D-PTSD)

Based on an earlier research in PTSD, Krystal and colleagues (1995) proposed that the thalamus plays an important role in dissociative-like states of altered consciousness. One of the functions of the thalamus is that of a sensory gate or filter, receiving direct and indirect input from sub-cortical areas (e.g., raphe nuclei and locus coeruleus), limbic regions (e.g., amygdala), and frontal areas (e.g., ACC and prefrontal cortices) [85]. Within this network, the thalamus may both directly and indirectly modulate responses to environmental stimuli, facilitating or impeding the flow of information [34, 61, 85].

Furthermore, the hippocampus and parahippocampal regions may be critical to the understanding of altered memory processing during dissociative states [31–33, 61].

Based on more recent neuroimaging findings in PTSD [10], Lanius and colleagues (2010) proposed a neurobiological model that distinguishes between two types of responses to traumatic reminders or other stressors: patients with a dissociative response type (D-PTSD) are thought to “over-modulate” their emotions, as opposed to patients who primarily suffer from re-experiencing symptoms, including (affective) hyperarousal, intense feelings of shame and guilt, and difficulties in emotion downregulation (re-experiencing response type). Emotion over-modulation (dissociative response type) is thought to primarily activate frontal regions implicated in cognitive control and emotion downregulation (e.g., dorsal/rostral ACC and mPFC), associated with dampened activity in amygdala and insula. The reversed pattern—diminished frontal recruitment (ACC and mPFC) and hyperactivity in amygdala and insula—is assumed to underlie emotion under-modulation (re-experience response type) [10].

Central to the development of this model [10] was a script-driven imagery fMRI study [57], in which PTSD patients were exposed to autobiographical narratives of traumatic events. The majority of patients (∼70%) reported marked re-experiencing symptoms and showed a substantial increase in heart rate during the script. In a smaller patient group (∼30%), however, this heart rate increase was not observed—instead, these patients showed stronger activity in the medial frontal gyrus, anterior and medial cingulate, middle temporal gyri (BA38), precuneus, occipital areas, and inferior frontal gyrus (IFG), compared to a control group of traumatized persons who had not developed PTSD [57].

In another fMRI study [86], patients with D-PTSD showed increased activity in the amygdala, insula, and thalamus while fearful versus neutral facial expressions were presented nonconsciously. Interestingly, these limbic(-related) areas were not significantly activated when images were presented consciously. In the latter case, dissociative patients showed increased activity in ventral PFC and diminished activity in the dorsomedial PFC, suggesting a conscious over-modulation of emotions and suppression of self-referential processing [86].

For PTSD patients who showed dissociative responses to autobiographical trauma scripts (compared to patients with a flashback response and healthy controls), there is also evidence for altered functional connectivity (FC), i.e., coactivation in areas implicated in sensory processing, consciousness, memory, and emotion regulation [56]: compared to controls, dissociative patients showed stronger FC of the left ventrolateral thalamus (VLT) with right insula, middle frontal gyrus, superior temporal gyrus, cuneus, and with left parietal lobe, but reduced VLT-FC with the left superior frontal gyrus, right parahippocampal gyrus, and right superior occipital gyrus. Compared to patients with a flashback response, dissociative patients showed increased FC between right cingulate gyrus and left IFG [56].

In the absence of experimental tasks, altered resting-state functional connectivity (RSFC) in the DMN was found in patients with D-PTSD [87•], including altered synchrony between the DMN (which is usually activated during rest) and the “central executive network” (commonly activated during cognitive tasks, as reflected in strong anticorrelations [75, 88]). Findings of altered intra-network resting-state connectivity (in DMN) and altered inter-network connectivity were significantly associated with depersonalization and derealization severity [87•].

In another RS-fMRI study [89•], patients with D-PTSD (compared to PTSD patients without the dissociative subtype and HC) demonstrated increased FC of the amygdala with prefrontal and parietal regions, including dorsal PCC and precuneus, which may further support the assumption of a distinct “neurobiological profile” of D-PTSD [89•].

#### Research in Dissociative Identity Disorder (DID)

There is some evidence that the aforementioned neurobiological alterations may not be specific to a specific disorder but rather represent a trans-diagnostic phenomenon related to dissociation. Recent findings in DID [90•, 91•] resemble findings for D-PTSD, albeit intra-individual differences (instead of inter-individual differences) were observed: neurobiological responses significantly differed depending on whether DID patients were in a “hyper-aroused traumatic identity state” (with voluntary access to traumatic memories) or in their “normal dissociative identity state” (characterized by dissociative amnesia) [90•, 91•]. In the two studies by Reinders and colleagues (2006, 2014), DID patients showed elevated cardiovascular responses (heart rate and blood pressure) and stronger amygdala and insula activity, along with lower activity in cingulate gyrus, parietal cortex, and parahippocampus when exposed to a trauma script (versus neutral script) while being in their “hyper-aroused traumatic identity state” than in their neutral “hypo-aroused identity state” [90•, 91•]. In another study, DID patients exhibited increased perfusion in bilateral thalamus while being in their (apparently) “normal” state of identity compared to an (apparently) “emotional” identity state [92•]. More research is needed to clarify whether brain activity patterns may differ dependent on states (in the same individuals) or represent stable inter-individual differences, which may allow for a discrimination between diagnostic subgroups/categories [10].

### Research on Structural Alterations

Aside from functional alterations, several studies reported structural abnormalities in clinical samples with high trait dissociation, although these structural findings are still quite heterogeneous.

In depersonalization disorder, reduced gray matter volumes (GMV) in right thalamus, caudate, and cuneus, and increased GMV in the left dorsomedial PFC and the right somato-sensoric regions were observed [93•]. As abovementioned, these areas have been implicated in dissociation [10, 61, 62, 85].

In DID, reduced volumes in the amygdala and hippocampus [94, 95] and parahippocampus [95] were found, although discrepant findings of normal amygdala and hippocampal volumes compared to healthy controls were also reported [96]. Smaller hippocampal volumes may be related to early life trauma: the hippocampus has a high density of glucocorticoid receptors and is highly sensitive to a heightened release of the stress hormone cortisol—therefore, chronic traumatic stress may lead to cell damage in this area [31–34]. Smaller hippocampal volumes were also found in healthy individuals with childhood trauma, who did not develop a disorder [97]. Reduced hippocampal volumes in PTSD [98•, 99] may therefore stem from a history of trauma rather than specific to the diagnosis [100]. In a recent study [98•], comparing PTSD patients with versus without dissociative subtype, no significant group differences in amygdala, hippocampus, and parahippocampus volumes were observed. Yet, patients with D-PTSD showed increased GMV in right precentral and fusiform gyri and reduced GMV in right inferior temporal gyrus. Severity of depersonalization and derealization was positively correlated with GMV in the right middle frontal gyrus [98•]. Another study in PTSD [101•] found positive associations between trait dissociation and GMVs in medial/lateral PFC, orbitofrontal, temporal polar, parahippocampal, and inferior parietal cortices—brain regions associated with emotion regulation.

Extending findings on GMV alterations, a recent study in dissociative disorders [102•] found significantly lower fractional anisotropy in white matter of the right anterior corona radiate (which receives projections from the basal ganglia) compared to healthy controls. More research is needed to understand how these alterations may be related to specific clinical symptoms of dissociation.

As already pointed out in the context of functional neuroimaging studies, interpretation of structural studies may be complicated by the presence of comorbidities. Patients with comorbid PTSD+DID showed significantly larger volumes of the putamen and pallidum than PTSD patients without DID [103]. Volumes of these regions (implicated in motor control [103–105]) were negatively correlated with severity of derealization/depersonalization [103]. Patients with PTSD+DID (but not PTSD patients without DID) further showed smaller hippocampal volumes than healthy controls [103].

Further studies with clinical control groups are needed to gain more insight into this relationship. Of note, structural alterations do not necessarily reflect functional alterations, i.e., more frequent engagement of specific brain areas does not have to be reflected in larger volume of this region and vice versa. Studies including both functional and structural measures may give additional insight into this relationship [106].

#### Interim Summary

In sum, theoretical assumptions and research in depersonalization/DDD, DID, and D-PTSD suggest a link between dissociative symptoms and alterations in brain regions associated with emotion processing and memory (amygdala, hippocampus, parahippocampal gyrus, and middle/superior temporal gyrus), attention and interoceptive awareness (insula), filtering of sensory input (thalamus), self-referential processes (PCC, precuneus, and mPFC), cognitive control, and arousal modulation (IFG, ACC, and lateral prefrontal cortices). As many studies did not include clinical control groups or groups of traumatized individuals who did not develop a disorder, it remains unclear whether the aforementioned results are related to a specific disorder or a trans-diagnostic feature, possibly associated with high dissociation. Findings that are based on correlations (e.g., between brain structure/function and psychometric scores) do not allow causal conclusions, i.e., whether they represent a predisposition for or a result of frequent dissociative experiences (see below).

The second aim of our article is to review neuroimaging studies in BPD that investigated links to dissociative symptoms. We searched databases (PubMed, PsychInfo, Web of Science, and Science Direct) for different combinations of “Borderline Personality Disorder,” “Dissociation,” and the following keywords: brain, brain alterations, functional magnetic resonance imaging, magnetic resonance imaging, neurobiological, neuroimaging, neurophysiological, positron emission tomography, and structural magnetic resonance imaging.

In the next section, we first describe clinical expressions of dissociation in BPD, providing a background for the subsequent discussion of neuroimaging research.

### Dissociation in Borderline Personality Disorder (BPD)

Transient stress-related dissociation is a hallmark of BPD [6, 11•, 107]. It has been defined as one of the nine diagnostic criteria for the disorder in DSM-IV [108]. In DSM-V [5], “dissociative states under stress” are still listed among other BPD key features such as emotion dysregulation, instable cognition, impulsivity, and interpersonal disturbances.

Emotion dysregulation in BPD (i.e., heightened sensitivity to emotional stimuli, intense emotions, rapid mood swings, and lack of functional emotion regulation strategies) can have detrimental effects on goal-directed behaviors in every-day life [6, 11•, 13, 109•]. Numerous studies suggest that a dysfunctional network of fronto-limbic brain regions, including a hyperreactivity of the amygdala and insula, and diminished recruitment of frontal regions (e.g., orbitofrontal cortex (OFC), mPFC, and dlPFC) during emotional challenge may underlie emotion dysregulation in BPD (e.g., see [110•, 111•, 112•]).

Stress-related dissociation occurs in about 75–80% of BPD patients [6, 113–118], typically lasting between minutes and hours, or days [119, 120]. The strength, frequency, and intensity of dissociative experiences are positively correlated to self-reported arousal/stress levels [6].

Research in BPD has found reasonably strong relationships between dissociation and childhood trauma, especially sexual abuse [118, 121–124], physical abuse, attachment difficulties, and parental neglect [118, 125, 126] (for an overview, see [11•]).

It has been proposed that stress-related dissociation in BPD may be a form of emotion modulation (e.g., increased attempts to inhibit emotions), comparable to observations in D-PTSD, especially in patients with severe childhood trauma [11•, 127]. By interfering with mental resources that are crucial to cognitive functioning [55, 60•], stress-related dissociation may hinder recovery [128]. BPD patients with high trait dissociation showed significant impairments across multiple neuropsychological domains (including memory, attention, and interference inhibition) [8] [129•]. Recent neuroimaging studies further suggest a substantial impact of experimentally induced dissociation on affective–cognitive functioning in BPD [55, 60•], as discussed in more detail below.

### Neuroimaging Research on Dissociation in BPD

To our knowledge, so far, only relatively few neuroimaging studies in BPD examined links between trait/state dissociation and brain function during resting state (RS) [130•, 131–134] or experimental tasks [135–140, 141•], and even fewer studies have directly investigated the impact of experimentally induced dissociation on neural processing [55, 59, 60•]. In the following section, we provide an overview of neuroimaging studies, revealed by our literature search, and recent unpublished research from our group. Table 1 gives an overview of these studies (*n* = 20), summarizing key results related to dissociation and methodological characteristics (sample characteristics, medication status, trauma history, comorbidity, and measures). In all studies, BPD was assessed according to DSM-IV [108].

**Table 1:** Overview of the studies on possible links between brain function, brain structure, and dissociation in borderline personality disorder (BPD)

Authors, year of publication	Groups (sample size), gender	Psychotropic medication status	Comorbidities and trauma history in the patient sample	Neuroimaging technique	Measures of dissociation (trait/state and time of assessment)	Key findings concerning dissociation
Hazlett et al. (2012)	• Groups: - BPD (*n* = 33) - Schizotypal PD (SPD; *n* = 28) - HC (*n* = 32) • Gender: mixed (female/male) BPD, 20/13; SPD, 12/16; and HC, 20/12	Medication-free for at least 6 weeks prior to scanning. Most patients (BPD, *n* = 16; SPD, *n* = 23) had never been medicated.	High rates of childhood abuse and neglect in BPD. Exclusion of history of schizophrenia, psychotic disorder, bipolar I, or current major depressive disorder (MDD). 21 BPD and 6 SPD had past MDD.	Event-related fMRI during processing of neutral, pleasant, and unpleasant pictures from the IAPS, each of which presented twice within their respective trial block/run.	Self-reported trait dissociation (DES)	BPD patients showed greater amygdala reactivity and prolonged amygdala activation to repeated emotional versus neutral IAPS pictures. Fewer dissociative symptoms in both patient groups were associated with greater amygdala activation to repeated unpleasant pictures.
Hoerst et al. (2010)	• Groups: - BPD (*n* = 30) - HC (*n* = 30) • Gender: female	Free of current psychotropic medication for at least 3 months prior to scanning procedure.	Some patients met criteria for current/lifetime posttraumatic stress disorder (PTSD; 11/13), MDD (3/18), substance abuse (0/7), as well as eating disorders and (other) anxiety disorders. Lifetime schizophrenia and bipolar I were excluded.	Proton magnetic resonance spectroscopy (MRS), to measure neurometabolic concentrations (glutamate levels) in the anterior cingulate cortex (ACC)	Self-reported trait dissociation (DES).	Significantly higher levels of glutamate in the ACC in patients with BPD as compared with healthy controls. Positive correlation between glutamate concentrations and dissociation as well as between glutamate concentration and subscores of the borderline symptom
Irle et al. (2007)	• Groups: - BPD (*n* = 30) - HC (*n* = 25) • Gender: female	8 patients were on antidepressant medication (SSRI) and 6 were occasionally treated with sedatives (e.g., benzodiazepine).	High rates of physical and sexual abuse in childhood and adolescence. Some patients met criteria for current/lifetime depersonalization disorder (27/27), dissociative amnesia (DA) (7/7), dissociative identity disorder (DID; 4/4), and PTSD (11/11).	Structural MRI to assess volumes of the superior (precuneus and postcentral gyrus) and inferior parietal cortices	Presence of comorbid dissociative disorders (SCID-D) and dissociative symptoms such as depersonalization and derealization (DIB)	BPD patients with comorbid DA or DID had significantly increased volumes of the left postcentral gyrus compared to healthy controls (+13%) and BPD patients without these disorders (+11%). In BPD subjects, stronger depersonalization was significantly correlated to larger right precuneus volumes.
Kluetsch et al. (2012)	• Groups: - BPD with history of self-harm (*n* = 25) - HC (*n* = 23) • Gender: female	Unmedicated sample	Some patients met criteria for current/lifetime PTSD (9/9), MDD (0/18), as well as eating disorders and (other) anxiety disorders, current MDD, substance abuse, and lifetime schizophrenia or bipolar I were excluded.	FMRI during painful heat versus neutral temperature stimulation (thermal sensory anlayzer II)	Self-reported trait dissociation (DES) and state dissociation, assessed prior to and immediately after scanning (DSS)	Higher self-reported trait dissociation was associated with an attenuated signal decrease of the default mode network in response to painful stimulation.
Kraus et al. (2009)	• Groups: - BPD with PTSD (*n* = 12) - BPD without co-occurring PTSD (*n* = 17) • Gender: female	Free of psychotropic medication for at least 2 weeks before scanning procedure.	12 BPD patients met criteria for current PTSD. Lifetime MDD (*n* = 21), eating disorders, and (other) anxiety disorders were present. Current MDD, substance abuse, and lifetime schizophrenia or bipolar I were excluded	FMRI during heat stimulation, assessed in five stimulation blocks (each for 30 s) with individually adapted temperature	Self-reported trait dissociation (DES) and state dissociation at the time of scanning (DSS)	Both groups of BPD patients did not differ significantly in pain sensitivity, while amygdala deactivation was more pronounced in BPD patients with co-occurring PTSD. Amygdala deactivation was independent of state dissociation.
Krause-Utz et al. (2014a)	• Groups: - BPD (*n* = 22) - HC (*n* = 22) • Gender: female	Medication-free for at least 14 days (in the case of fluoxetine, 28 days) prior to scanning procedure.	All BPD patients had a history of childhood abuse/interpersonal trauma. Some patients met criteria for current/lifetime PTSD (9/11), other anxiety, and eating disorders. Current MDD, substance abuse (6 months prior to scan) and lifetime schizophrenia or bipolar I were excluded	Event-related fMRI during performance of an emotional working memory task (EWMT, adapted Sternberg item recognition task) with negative versus neutral interpersonal IAPS pictures	Self-reported trait dissociation (DES) and state dissociation immediately before and after scanning (DSS4).	In the BPD group, increase of self-reported dissociative states (DSS4 scores) over the course of the EWMT positively predicted bilateral amygdala connectivity with and left insula, left precentral gyrus, right thalamus, and right anterior cingulate during emotional distraction.
Krause-Utz et al. (2012)	• Groups: - BPD (*n* = 22) - HC (*n* = 22) • Gender: female	Medication-free for at least 14 days (in the case of fluoxetine, 28 days) prior to scanning procedure.	All BPD patients had a history of interpersonal trauma (including severe childhood abuse/neglect). Some patients met criteria for current/lifetime PTSD (9/11). Current MDD, substance abuse, and lifetime schizophrenia or bipolar I were excluded.	Event-related fMRI during performance of an emotional working memory task (EWMT, adapted Sternberg item recognition task) with negative versus neutral interpersonal IAPS pictures.	Self-reported trait dissociation (DES) and state dissociation immediately before and after scanning (DSS4)	In the BPD group, increase of self-reported dissociative states (DSS4 scores) over the course of the EWMT negatively predicted bilateral amygdala activity during emotional distraction.
Krause-Utz et al. (2015)	• Groups: - BPD (*n* = 27) - HC (*n* = 26) • Gender: female	Free of psychotropic medication at least 4 weeks before scanning.	Some patients met criteria for current/lifetime PTSD (14/18), other anxiety disorders. and eating disorders. Lifetime diagnosis of psychotic disorder, bipolar I disorder, and alcohol/substance abuse 6 months prior to scan were excluded.	FMRI during a differential delay aversive conditioning paradigm with an electric shock as unconditioned stimulus and two neutral pictures as conditioned stimuli (CS+ and CS−)	Self-reported trait dissociation (DES) and state dissociation before and after scan (DSS)	Amygdala habituation to CS+^paired^ (CS+ in temporal contingency with the aversive event) during acquisition was found in HC, but not in patients. No significant correlations with dissociative symptoms.
Krause-Utz et al. (2014b)	• Groups: - BPD (*n* = 20) - HC (*n* = 17) • Gender: female	Medication-free for at least 14 days (in the case of fluoxetine, 28 days) prior to scanning procedure.	All BPD patients had a history of interpersonal trauma. Some patients met criteria for current PTSD (*n* = 9), other anxiety disorders, and eating disorders. Current MDD, substance abuse (6 months prior to scan), and lifetime schizophrenia or bipolar I were excluded.	Resting state (RS) fMRI was acquired to investigate RS functional connectivity in the medial temporal lobe network (seed: amygdala), salience network (seed: dorsal ACC), and default mode network (seed: ventral ACC).	Self-reported trait dissociation (DES)	Self-reported trait dissociation positively predicted amygdala connectivity with dorsolateral prefrontal cortex and negatively predicted amygdala connectivity with during resting state. No differences were found between BPD with or without comorbid PTSD.
Krause-Utz et al. (submitted)	• Groups: - BPD patients exposed to a dissociation script (BPDd: *n* = 17) - Patients exposed to neutral script (BPDn: *n* = 12) - HC (*n* = 18) • Gender: female	Free of psychotropic medication for at least 4 weeks prior to the study.	All patients reported at least one type of severe to extreme childhood trauma. 5 patients in the BPDn group and 7 in the BPDd group met criteria for current PTSD. Comorbidity with current (other) anxiety and eating disorders was evident. Lifetime psychotic disorder, bipolar I disorder, mental retardation, and alcohol/substance abuse 6 months prior to scan were excluded.	FMRI to measure changes in BOLD signal, combining script-driven imagery (to experimentally induce dissociation) with a subsequent EWMT (to investigate working memory performance during emotional distraction)	Dissociation was induced in 17 patients, while 12 patients and 18 HC were exposed to a personalized neutral script. Self-reported trait dissociation (DES) and state dissociation at baseline, after script and after EWMT	BPDd showed overall WM impairments, significantly reduced bilateral amygdala activity (across conditions) and reduced left cuneus, lingual gyrus, and posterior cingulate activity (during negative distractors) compared to BPDn. Inferior frontal gyrus activity was higher in both BPD groups than in HC. BPDd further showed a stronger coupling of amygdala with right superior/middle temporal gyrus, right middle occipital gyrus, left inferior parietal lobule, and left claustrum than BPDn and HC.
Lange et al. (2005)	• Groups: - BPD (*n* = 17) - HC (*n* = 9) • Gender: female	5 BPD patients were on antidepressant medication and some were occasionally treated with benzodiazepines (*n* = 4) or mild neuroleptics (*n* = 3).	All BPD participants had experienced severe childhood sexual and physical abuse. Some patients met criteria for current PTSD (*n* = 6), depersonalization disorder (*n* = 14), DA (*n* = 4), and DID (*n* = 1). Nearly all patients met criteria for current or lifetime MDD (*n* = 16).	18Fluoro-2-deoxyglucose positron emission tomography (FDG-PET) to assess glucose metabolism in temporo-parietal cortices	Presence of comorbid dissociative disorders (SCID-D) and self-reported trait dissociation (DES)	BPD patients demonstrated reduced FDG uptake in the right temporal pole/anterior fusiform gyrus and in the left precuneus and posterior cingulate cortex. Impaired memory performance among borderline subjects was significantly correlated with metabolic activity in ventromedial and lateral temporal cortices.
Ludascher et al. (2010)	• Group: BPD patients (*n* = 15) • Gender: female	Medication-free for at least 14 days (in the case of fluoxetine, 28 days) prior to scanning procedure.	10 BPD patients had comorbid PTSD following severe childhood abuse (6 patients reporting sexual abuse, 3 patients reporting physical abuse, and 1 patient reporting neglect). Some patients met criteria for current/lifetime other anxiety and eating disorders. Lifetime diagnosis of psychotic disorder, bipolar I disorder, and alcohol/substance abuse 6 months prior to scan were excluded.	FMRI to measure changes in BOLD signal during script-driven imagery: participants were exposed to a personalized dissociative-inducing script (versus a neutral script) during the fMRI scan.	Self-reported trait dissociation (DES) and state dissociation before and immediately after script inside the MRI scanner (DSS-4)	BPD patients showed a significant increase in the left inferior frontal gyrus during the dissociation script. DSS4 ratings positively predicted activity in the left superior frontal gyrus and negatively predicted activity in the right middle and inferior temporal gyrus. In BPD patients with comorbid PTSD, increased activity in the left cingulate gyrus was found during the dissociation script. In this subgroup, DSS4 positively predicted bilateral insula activity and negatively predicted activity in the right parahippocampal gyrus.
Niedtfeld et al. (2013)	• Groups: - BPD (*n* = 60) - HC (*n* = 60) • Gender: female	Free of psychotropic medication	21 of the BPD patients met criteria for current PTSD. Traumatic events included severe physical and sexual childhood abuse.	Structural MRI to assess anatomical scans. Whole-brain gray matter volumes (GMV) were studied using voxel-based morphometry (VBM).	Self-reported trait dissociation (DES); scores were available in 42 BPD patients.	Trait dissociation (DES score) was positively correlated to GMV in the middle temporal gyrus in BPD.
Paret et al. (2016)	• Group: BPD patients (*n* = 8) • Gender: female	All participants were on stable medication.		Real-time fMRI-based neurofeedback training comprising of four sessions, in which participants viewed aversive images and received feedback from a thermometer displaying amygdala BOLD signals. Amygdala activity and functional connectivity were studied.	Self-reported state dissociation (DSS4) at the end of each run	Task-related amygdala-ventromedial prefrontal cortex connectivity was altered across the four sessions, with an increased connectivity during instructed emotion regulation versus viewing emotional pictures without regulation. Self-reported state dissociation and “lack of emotional awareness” decreased with training.
Rusch et al. (2007)	• Groups: - BPD and comorbid attention deficit hyperactivity disorder (ADHD; *n* = 20) - HC (*n* = 20) • Gender: female	Medication-free (including methylphenidate) for at least 2 weeks prior to scanning procedure	5 patients met criteria for current PTSD, 10 patients reported a history of sexual abuse in childhood, and 14 patients met criteria for past MDD. Exclusion of current major depression, substance abuse 6 months prior to study, lifetime substance dependence, schizophrenia, and bipolar I.	Diffusion tensor imaging (DTI) was used to measure mean diffusivity and fractional anisotropy in the inferior frontal white matter	Self-reported trait dissociation (DES)	Patients showed increased mean diffusivity in inferior frontal white matter, which was associated with higher levels of dissociative symptoms, dysfunctional affect regulation, anger-hostility, and general psychopathology but not associated with a history of sexual abuse.
Sar et al. (2007)	• Groups: - DID (*n* = 21, 15 with BPD) - HC without history of child abuse and trauma (*n* = 9) • Gender: mixed (female/male) Patients, 14/7 HC, 6/3	Medication-free for at least 1 month prior to study	All patients reported at least one type of severe childhood abuse and/or neglect, 15 patients had comorbid BPD+DID, and 6 patients had DID without BPD.	Single photon emission computed tomography (SPECT) with Tc99m-hexamethylpropylenamine (HMPAO) as a tracer was used to measure regional cerebral blood flow.	Self-reported trait dissociation (DES)	Patients showed decreased reduced cerebral blood flow in the orbitofrontal cortex (OFC) and occipital regions bilaterally. There was no significant correlation between rCBF ratios of the regions of interest and self-reported dissociation.
Wingenfeld et al. (2009)	• Groups: - BPD (*n* = 20) - HC (*n* = 20) • Gender: mixed (14 females and 6 males in each group)	12 of the BPD samples received psychotropic medication (including antidepressants and neuroleptics).	17 BPD patients reported at least mild PTSD symptoms and 5 patients fulfilled criteria for current PTSD. Some patients met criteria for current MDD (*n* = 3), bulimia nervosa (*n* = 3), social phobia (*n* = 1), and somatoform disorder (*n* = 1). Current MDD with psychotic symptoms, schizophrenia, schizoaffective disorders, anorexia, and substance dependence 6 months prior to the study were excluded.	fMRI to measure changes in BOLD signal during performance of an individualized emotional stroop task (EST), with neutral, general negative words, and individual negative words (selected from a prior interview with each participant).	Self-reported state dissociation before and after scanning (DSS21 akut) as well as dissociation within the past 7 days (DSS21)	Overall BPD patients had slower reaction times, which however were not correlated with dissociation. Healthy controls—but not BPD patients—showed significant recruitment of the ACC for negative versus neutral and individual negative versus neutral conditions, respectively. No significant correlations between DSS scores and BOLD signal were reported.
Winter et al. (2015)	• Groups: - BPD patients exposed to a dissociation script: BPDd (*n* = 18) - Patients exposed to neutral script: BPDn (*n* = 19) - HC (*n* = 19) • Gender: female	Free of psychotropic medication for at least 4 weeks prior to the study.	7 patients in the BPDn group and 8 in the BPDd group met criteria for current PTSD. Comorbidity with current (other) anxiety and eating disorders was evident. Lifetime psychotic disorder, bipolar I disorder, mental retardation, and alcohol/substance abuse 6 months prior to scan were excluded	fMRI to measure changes in BOLD signal, combining script-driven imagery (to experimentally induce dissociation) with a subsequent emotional stroop task (EST; containing negative, neutral, and positive words—to measure cognitive control of emotional material).	Dissociation was induced in 18 patients, while 19 patients and 19 HC were exposed to a personalized neutral script. Self-reported trait dissociation (DES) and state dissociation at baseline, after script (twice before EST, twice within EST, and once after EST)	BPD patients after dissociation induction (BPDd) showed overall slower and less accurate responses as well as longer reaction times for negative versus neutral words than BPDn. Moreover, BPDd showed increased activity in the inferior frontal gyrus and dorsolateral prefrontal cortex (dlPFC) during negative than neutral words. BPDn patients showed increased activity in the right superior temporal gyrus for emotional (positive and negative) versus neutral words compared to HC.
Wolf et al. (2011)	• Groups: - BPD (*n* = 17) - HC (*n* = 17) • Gender: female	All BPD patients were on stable medication for at least 2 weeks before scanning (psychotropic medication included antidepressants, mood stabilizers, and antipsychotics).	Some patients met criteria for current/lifetime MDD (9/5), past substance abuse (*n* = 6), eating disorders, and anxiety disorders other than PTSD. Current PTSD, lifetime schizophrenia, bipolar disorder, ADHD, and substance abuse 6 months prior to study were excluded.	RS-fMRI was acquired to investigate RS functional connectivity in large-scale brain networks.	Self-reported dissociation (DSS)	Self-reported state dissociation and tension (DSS) positively predicted RS functional connectivity of the insula and precuneus in the BPD group.
Wolf et al. (2012)	• Groups: - BPD (*n* = 16) - HC (*n* = 16) • Gender: female	All BPD patients were on stable medication for at least 2 weeks before scanning (psychotropic medication included antidepressants, mood stabilizers, and antipsychotics).	Some patients met criteria for current/lifetime MDD (8/5), past substance abuse (*n* = 6), eating disorders, and anxiety disorders other than PTSD. Current PTSD, lifetime schizophrenia, bipolar disorder, ADHD, and substance abuse within 6 months prior to study were excluded.	Continuous arterial spin labeling magnetic resonance imaging	Self-reported dissociation (DSS)	Compared to controls, BPD patients exhibited decreased blood flow in the medial OFC, whereas increased blood flow was found in the left and right lateral OFC. Correlation analyses revealed a positive relationship between medial and lateral orbitofrontal blood flow and impulsivity Barrett impulsiveness scale (BIS), but not with dissociation (DSS).

Note: *ADHD* attention deficit hyperactivity disorder, *BOLD* blood oxygen level-dependent, *BPD* borderline personality disorder, *CTQ* childhood trauma questionnaire, *DA* dissociative amnesia, *DID* dissociative identity disorder, *DES* dissociation experience scale, *DSS4* dissociaton stress scale 4-item version, *DIB* diagnostic interview for borderline patients, *DSS21* dissociation stress scale 21-item version, *DSM-IV* Diagnostic and Statistical Manual of Mental Disorders, fourth edition, *EST* emotional stroop task, *fMRI* functional magnetic resonance imaging, *GMV* gray matter volume, *HC* healthy controls, *IAPS* International Affective Picture System, *MDD* major depressive disorder, *OFC* orbitofrontal cortex, *PTSD* posttraumatic stress disorder, *RS* resting state, *SCID-D* structural clinical interview for DSM-IV dissociative disorders, *SPD* schizotypal personality disorder, *SSRI* selective serotonin reuptake inhibitor, *VBM* voxel-based morphometry

#### Brain Function During Rest: PET, SPECT, and RS-fMRI Studies

Lange and colleagues used 18fluoro-2-deoxyglucose (FDG-)PET to investigate glucose metabolism in 17 BPD patients (with a history of childhood sexual/physical abuse, mixed gender, partly medicated; see Table 1) and 9 healthy controls (HCs) [131]. BPD patients displayed reduced FDG uptake in the right temporal pole, anterior fusiform gyrus, and left precuneus and PCC. Impaired memory performance among patients was correlated with metabolic activity in ventromedial and lateral temporal cortices (implicated in episodic memory consolidation and retrieval), while no correlations with trait dissociation (DES) were reported. The finding of decreased temporo-parietal metabolism was discussed as possible neural underpinning of altered memory processes that may also play a role in the context of dissociation [131]. However, sample sizes were relatively small, and findings may not be specific to BPD due to comorbidities (depersonalization disorder, DID, and PTSD).

Sar et al. [132] used single-photon emission computed tomography (SPECT) with Tc99m-hexamethylpropylenamine (HMPAO) as a tracer to investigate regional cerebral blood flow (rCBF) in an unmedicated sample of DID patients (*n* = 21), 15 of whom met criteria for comorbid BPD, and 9 HCs. Compared to HCs, patients showed decreased rCBF ratio in bilateral medial OFC and increased rCBF in medial/superior frontal regions and occipital regions bilaterally [63]. No significant correlation with dissociation was reported.

Wolf and colleagues (2012) used continuous arterial spin labeling MRI to measure alterations in blood flow in 16 female BPD patients without comorbid PTSD (partly medicated but on a stable medication) and 16 HCs [133]. Compared to HCs, patients exhibited decreased blow flow in the medial OFC, while increased blood flow was found in the lateral OFC bilaterally. Like in the study by Sar and colleagues [132], no significant correlation with self-reported dissociation (DSS) was observed. Instead, medial and lateral OFC blood flow was positively correlated with impulsivity, as measured with the Barratt impulsiveness scale (BIS; [142])—suggesting an association with impulsivity rather than with dissociation.

In 17 female BPD patients without PTSD and 17 HCs (same sample as in [133]), Wolf and colleagues (2011) investigated changes in RSFC using RS-fMRI [134]. Within the DMN, patients showed increased RSFC in the left frontopolar cortex and insula, while showing decreased RSFC in the left cuneus. Within a fronto-parietal network, patients exhibited decreased RSFC in the left inferior parietal lobule and the right middle temporal cortex compared to HCs. No significant group differences were observed in two other networks comprising lateral prefrontal and cingulate regions. In the BPD group, state dissociation (DSS) positively predicted RSFC in the insula and precuneus [134]—as previously mentioned, these regions play a role in interoceptive awareness and self- referential processes [74].

In another RS-fMRI study, Krause-Utz et al. (2014) investigated the changes in RSFC of the three fronto-limbic core regions that are of specific relevance to BPD: bilateral amygdala (medial temporal lobe network), bilateral dorsal ACC (salience network), and bilateral ventral ACC (default mode network) [130•]. The DES was used to predict RSFC with these seed regions. The sample comprised 20 unmedicated female BPD patients (all with a history of interpersonal trauma) and 17 HCs. There was a trend for increased amygdala RSFC with right dorsal insula, lateral OFC, and putamen in patients compared to HCs. BPD patients further exhibited diminished negative RSFC between the dorsal ACC and the left PCC and increased negative RSFC of the left ventral ACC with occipital cortex, lingual gyrus, and cuneus. In the patient group, DES scores positively predicted amygdala RSFC with the right dlPFC. Furthermore, patients, who reported higher trait dissociation showed a reduced coupling of the amygdala with right fusiform gyrus. A subgroup comparison of BPD patients with (*n* = 9) versus without comorbid PTSD (*n* = 11) yielded similar findings.

The two RS-fMRI studies described above [130•, 134] provide primary evidence for a possible role of dissociation in altered RSFC in BPD. As pointed out before, no causal conclusions can be drawn from correlational findings. In order to gain more insight into the impact of dissociation on RSFC of large-scale brain networks in BPD, future studies may acquire resting-state scans before and after experimentally inducing dissociation (e.g., via script-driven imagery) and before and after therapeutic interventions aimed at a reduction of dissociative symptoms.

Again, interpretation of findings, summarized so far, is complicated by methodical differences (e.g., comorbidities with depersonalization disorder [131], DID [131, 132], and PTSD [130•]), trauma history [130•, 131], psychotropic medication [131, 133, 134], and relatively small sample sizes.

#### Neurochemical Alterations: MRS Studies

To our knowledge, one MRS study in BPD investigated links between trait dissociation (DES) and glutamate levels in the ACC within a sample of unmedicated female BPD patients (*n* = 30) and HCs (*n* = 30) [143]. Significantly higher levels of glutamate in the ACC were found in BPD as compared with HCs. In BPD, glutamate concentrations in the ACC were positively correlated not only to DES scores, but also to BIS scores. Associations between ACC glutamate levels and impulsivity (BIS scores) could be replicated in a more recent study [144], suggesting a link with impulsivity rather than with dissociation.

#### Task-Related fMRI Studies

A couple of fMRI studies in BPD examined links between changes in BOLD response during experimental tasks (e.g., viewing aversive images, pain stimulation, and cognitive tasks) and self-reported dissociation (e.g., DSS and DES).

Kraus and colleagues (2009) investigated amygdala activity in relation to pain processing (heat stimulation) and state dissociation (DSS) in an unmedicated sample of female BPD patients with (*n* = 12) and without comorbid PTSD (*n* = 17) [137]. No significant group differences in pain sensitivity were observed. A deactivation of the amygdala during pain processing was found to be more pronounced in BPD patients with comorbid PTSD than in those without PTSD, while this was not significantly correlated with DSS scores.

In a more recent study, pain processing and dissociation (DES and DSS before/after scanning) were investigated in relation to FC changes in an unmedicated female sample of BPD patients (*n* = 25, all with a history of self-harm) and 23 HCs [136]. Kluetsch and colleagues [136] used psychophysiological interaction (PPI) analysis [136] and independent component analysis to analyze changes in FC. Compared to controls, patients showed a reduced integration of the left retrosplenial cortex and left superior frontal gyrus into the DMN. During pain versus neutral temperature stimulation, patients further exhibited less FC between the PCC (seed region) and left dlPFC. Higher trait dissociation (DES) was associated with an attenuated signal decrease of the DMN in response to painful stimulation. Since only patients with a history of self-harm and no clinical control group were included, future studies should clarify whether these findings can be replicated in BPD patients without self-injurious behavior and whether they may underlie altered pain processing (e.g., analgesia) during dissociation.

Wingenfeld and colleagues (2009) applied an individualized emotional stroop task (EST, with neutral, general negative, and individual negative words) to investigate changes in BOLD signal during emotional interference in 20 BPD patients (partly medicated, mixed gender; see Table 1) and 20 HCs [140]. State dissociation (DSS) was assessed before and after scanning. Patients displayed overall slower reaction times than HCs, while no increase of reaction times after emotional interference was observed. Controls, but not BPD patients, showed a significant recruitment of the ACC and frontal areas for generally negative versus neutral and for individual negative versus neutral words, respectively. No significant correlations between DSS and behavioral measures or neural activity were reported.

Hazlett and colleagues (2012) investigated potentiated amygdala responses to repeatedly presented emotional pictures in an unmedicated sample of BPD patients (*n* = 33), schizotypal personality disorder (SPD) patients (*n* = 28), and 32 HCs (mixed gender; see Table 1) [135]. Participants underwent event-related fMRI scanning while viewing repeated (versus novel) neutral, pleasant, and unpleasant pictures. BPD patients demonstrated increased amygdala reactivity to repeated emotional, but not neutral pictures, and a prolonged return to baseline of amygdala activity across all conditions. Despite amygdala overactivation, BPD patients showed blunted arousal ratings of emotional, but not neutral pictures. A significant negative correlation between self-reported dissociation and amygdala activity was found in BPD and also in the SPD group: higher self-reported dissociation (DES) was associated with lower emotion-challenged amygdala reactivity. This latter finding is in line with the assumption that dissociation may serve as a defensive mechanism for unpleasant stimuli (see above).

The influence of dissociation on brain activity and FC during emotional distraction in the context of a working memory task was investigated in two fMRI studies by Krause-Utz and colleagues [139, 145•]. Twenty-two unmedicated female BPD patients (all with a history of interpersonal trauma) and 22 HCs performed a modified Sternberg item-recognition task (emotional working memory task, EWMT, with neutral versus negative interpersonal pictures versus no distractors presented during the delay interval between encoding and retrieval). Immediately before and after the EWMT, state dissociation (DSS) was measured. Patients showed significantly increased amygdala activation and impaired task performance during distraction by negative and neutral interpersonal pictures but not in the control condition without distractors, suggesting increased susceptibility to social cues in BPD [139]. Patients who reported a stronger increase of state dissociation (DSS) showed significantly lower amygdala reactivity to negative distractors [139], in line with aforementioned findings by Hazlett and colleagues [135] and theoretical models [10]. No significant differences were observed between BPD patients with (*n* = 9) versus without comorbid PTSD.

In a reanalysis of this data set, Krause-Utz, Elzinga, and colleagues (2014) further examined how activity in the bilateral amygdala (seed region) was correlated to activity in other regions across the brain [145•] using PPI [146]. In the BPD group, a stronger coupling of the amygdala with hippocampus and dorsomedial PFC was observed for negative versus no distractors, suggesting an increased coactivation (information exchange) of regions involved in emotional and self-referential processing, e.g., retrieval of autobiographical memories [145•]. During negative distractors, patients with higher state dissociation (DSS4) showed stronger bilateral amygdala FC with right thalamus, right ACC, left insula, and left precentral gyrus—regions implicated in arousal modulation, body movements, attention, and filtering of sensory information [145•].

In another fMRI study, Krause-Utz, Mauchnik, and colleagues [138] investigated the neural correlates of classical fear conditioning (using a differential delay aversive conditioning paradigm with an electric shock as unconditioned stimulus and two neutral pictures as CS− and CS+) in 27 unmedicated female BPD patients and 26 HCs. Controls but not BPD patients showed amygdala habituation during acquisition of CS+^paired^ (CS+ in temporal contingency with the aversive event). No significant effect of trait or state dissociation (DSS) was observed, neither on skin conductance response (SCR) nor on brain activity. In contrast to this, an earlier study by Ebner-Priemer and colleagues (2009) found diminished fear conditioning in terms of SCR and subjective ratings in BPD patients with high (compared to those with low) pre-experimental dissociation [7]. These discrepancies may be related to methodological differences (e.g., assessment of skin conductance during fMRI versus in the laboratory and different sample characteristics: patients in the study by Krause-Utz et al. [106] reported relatively low naturally occurring state dissociation).

#### Script-Driven Imagery Studies

To our knowledge, three fMRI studies in BPD so far have used script-driven imagery to experimentally induce dissociative states [55, 59, 60•].

In a pilot fMRI study, Ludascher and colleagues [120] investigated dissociation and pain sensitivity in unmedicated female BPD patients with (*n* = 10) and without comorbid PTSD (*n* = 5) [59]. During fMRI, patients were exposed to an autographical dissociation script versus a neutral script. After the dissociation script, DSS scores were significantly increased, indicating a successful experimental manipulation, and pain sensitivity was lower than after the neutral script. During the dissociation script, patients further showed increased activation in the left IFG (BA9). Scores on the DSS positively predicted activation in the left superior frontal gyrus (BA6) and negatively predicted activation in the right middle temporal gyrus (BA21) and inferior temporal gyrus (BA20). In the subgroup of patients with BPD+PTSD (*n* = 10), increased activity in the left cingulate gyrus (BA32) was observed during the dissociation script. Further, DSS scores were positively correlated to the bilateral insula activity (BA13) and negatively correlated with the right parahippocampal gyrus (BA35) activity. However, the sample size was relatively small and no control group was included.

In a subsequent study, Winter and colleagues (2015) investigated the impact of dissociation on cognitive control of emotional material, by combining script-driven imagery with an EST (with negative, neutral, and positive words), a free word recall task, and a recognition task for EST words [60•]. Dissociation was induced in 19 unmediated female BPD patients (BPDd), while 18 BPD patients (BPDn) and 19 HCs were exposed to a neutral script. Script-driven imagery again successfully increased dissociation (DSS) in the BPDd group. During the EST, BPDd patients showed overall cognitive impairments (more errors and slower reaction times) and prolonged reaction times in response to negative versus neutral words compared with the other groups. In the subsequent memory task, the BPDd group recalled fewer EST words and needed more time to recognize words than BPDn. Further, BPDd demonstrated increased activity in left IFG and dlPFC during negative versus neutral words, possibly reflecting increased attempts to inhibit (emotional) material during dissociation.

Recently, Krause-Utz and colleagues (submitted) combined script-driven imagery with the EWMT to study the effect of dissociation on amygdala activity and FC during emotional distraction [55]. Within an unmedicated female sample of 29 BPD patients, dissociation was induced in 17 patients (BPDd), while 12 patients (BPDn) and 18 HC were exposed to a neutral script. Script-driven imagery again successfully increased dissociation (DSS) and aversive pictures within the EWMT evoked significant changes in amygdala activity. Overall, the BPDd patients showed significantly more working memory impairments (incorrect responses and misses, suggesting disturbed memory encoding/retrieval), and reduced activity in bilateral amygdala. Amygdala hyperactivity to emotional pictures (relative to HCs) could only be observed in BPDn. During negative distractors (versus no distractors), BPDd patients further showed lower activity in left cuneus, lingual gyrus, and PCC than BPDn and increased left IFG activity than HC. Increased left IFG activity in BPDd resembles findings of previous studies [59, 60•], albeit it was also found in the BPDn group [55]. The PPI revealed a significantly stronger coupling of amygdala with the left inferior parietal lobule, right superior/middle temporal gyrus, right middle occipital gyrus, and left claustrum during negative distractors in the BPDd group compared to the other groups [55]. More studies, including clinical control groups and larger samples, are needed to gain a deeper insight into the impact of dissociation on neural processes during affective–cognitive processing.

#### Possible Effects of fMRI Neurofeedback

Paret and colleagues [141•] used real-time fMRI to investigate the effects of a neurofeedback training task on amygdala activity and amygdala-PFC FC. In four training sessions, female BPD patients (*n* = 8) were instructed to downregulate emotional responses to aversive images based on feedback from a thermometer display, showing amygdala BOLD signals [141•]. The DSS was applied at the end of each training run. Amygdala-PFC FC was altered across the four sessions, with increased amygdala-vlPFC FC for regulating versus passively viewing aversive pictures. Interestingly, self-reported “lack of emotional awareness” (difficulties in emotion regulation scale [147]) and also state dissociation (DSS) decreased with training [148].

### Structural Neuroimaging Studies in BPD

A large number of neuroimaging studies in BPD used techniques like structural MRI or DTI to investigate volumetric alterations in comparison to HCs (e.g., see [110•, 112•]). Some of these studies included psychometric scales like the DES or DSS to link their findings to dissociation [149, 150•, 151].

Irle et al. [149] used structural MRI to investigate volumes of the superior (precuneus and postcentral gyrus) and inferior parietal cortices in 30 female BPD patients (all with a history of severe sexual and physical abuse, partly medicated; see Table 1), and 25 HCs. Comorbid dissociative disorders were determined using the structured clinical interview for DSM-IV dissociative disorders (SCID-D) [152]. BPD patients with comorbid dissociative amnesia (DA) or DID had significantly increased left postcentral gyrus volumes compared to HCs (+13%) and BPD patients without these disorders (+11%). In the entire BPD sample, smaller right-sided precuneus volumes (−9%) were observed (compared to HCs) and stronger depersonalization was related to larger right precuneus size, suggesting a possible relationship between dissociative symptoms and volumetric alterations in this region.

Niedtfeld and colleagues (2013) used voxel-based morphometry to investigate structural alterations across the entire brain in a female sample of 60 unmedicated BPD patients with (*n* = 21) and without comorbid PTSD (*n* = 31; mainly following severe childhood trauma such as physical/sexual abuse), and HCs (*n* = 60) [150•]. In BPD, smaller GMV of right amygdala, hippocampus, ACC, fusiform, and inferior temporal gyrus were found. In patients with comorbid BPD+PTSD, increased GMV in the dlPFC and superior temporal gyrus were observed. For a smaller subsample of 42 BPD patients, scores on the DES could be included, which predicted larger GMV in the middle temporal gyrus [150•]—a region previously implicated in dissociation [83, 84].

Extending the abovementioned findings on GMV alterations, Rusch and colleagues (2007) used DTI to study white matter alterations in 20 female unmedicated BPD patients and 20 HCs [151]. Mean diffusivity in inferior frontal white matter was associated with higher trait dissociation (DES), but also with measures of general psychopathology. As all BPD patients had comorbid attention deficit hyperactivity disorder (ADHD), it also remains unclear whether these alterations are specific for BPD.

#### Interim Summary

**Fig. 1:**
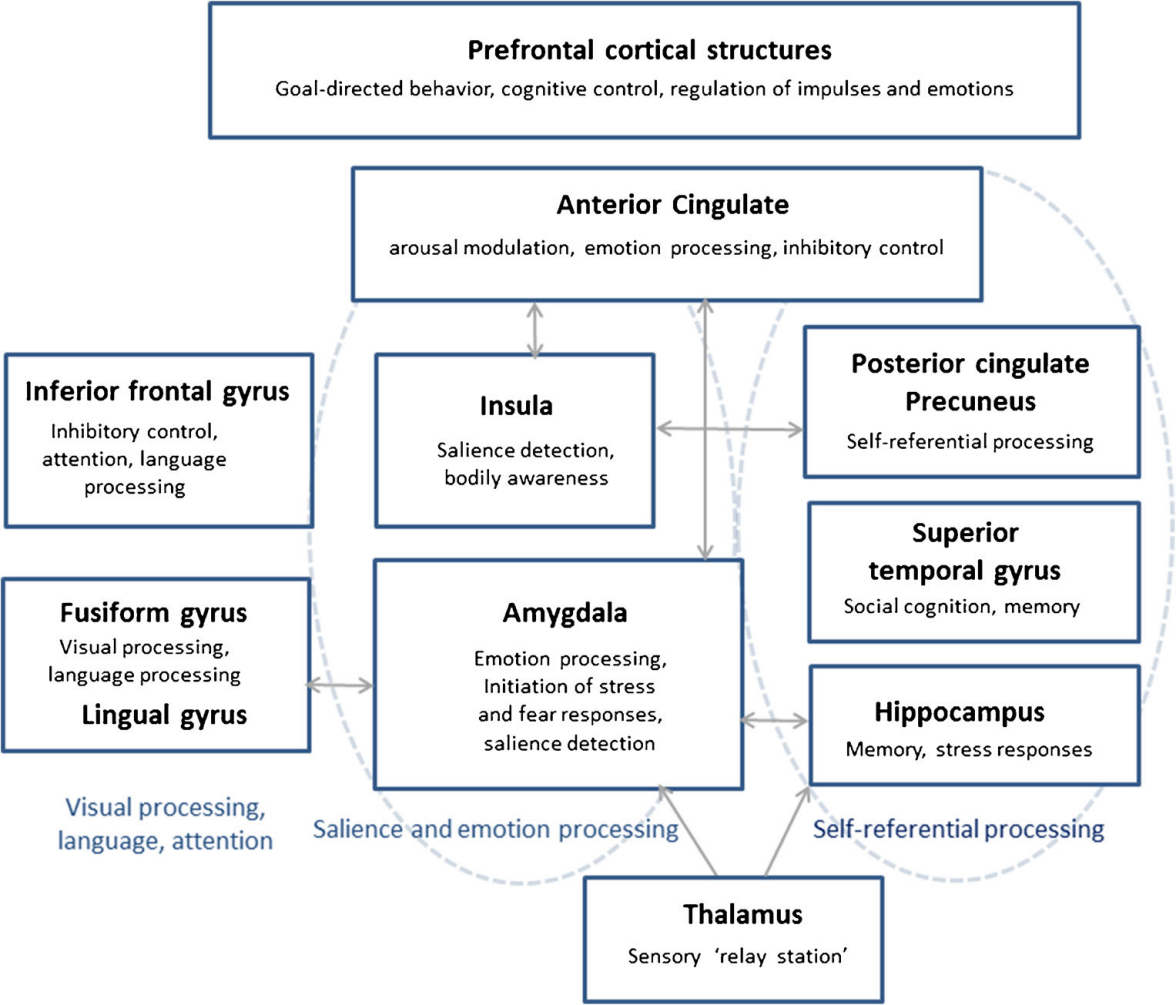
A schematic overview of brain regions and functions (among others) associated with dissociation in borderline personality disorder. The precise neurobiological underpinnings of dissociation remain elusive, but there is evidence for a link between dissociative states/traits and altered (co)activity in brain networks involved in emotion processing and memory (e.g., amygdala and hippocampus/medial temporal lobe memory system), interoception and attention regulation (insula), self-referential processes (e.g., posterior cingulate cortex and precuneus), cognitive control, and arousal modulation (e.g., dorsolateral prefrontal cortex (dlPFC), inferior frontal gyrus, and anterior cingulate cortex)—functions which may be altered during dissociation

To sum up at this point, structural studies on dissociation in BPD are still relatively rare and heterogeneous. Task-related fMRI studies suggest a role of dissociation in brain regions that play an important role in emotion processing/regulation, pain processing, and impulse control, including the amygdala, medial temporal lobe, insula, fusiform gyrus, precuneus, IFG, ACC, and cortical structures (e,g., dlPFC) [110•, 111•, 112•]. These brain regions and (part of) their functions are summarized in Fig. 1.

As previously mentioned, the amygdala is fundamentally involved in emotion processing and the initiation of stress and fear responses [63, 67–69] and activity in this area might be dampened during dissociation [10, 62]. Three studies in BPD [55, 135, 139] found lower activity in this region in patients reporting higher dissociation, while other studies did not [60•, 137, 138]. These discrepancies may partly be related to differences in study design: studies observing negative correlations studied amygdala reactivity to highly aversive (trauma-related) pictures [55, 135, 139], while Kraus and colleagues [137] studied amygdala activation during pain stimulation and Winter et al. [60•] used emotional words, which might have been less distressing than aversive pictures (not evoking significant changes in amygdala activity). Naturally occurring dissociative states (DSS4 ratings: mean ± standard deviation (M ± SD)) were relatively low ([137]: BPD 1.16 ± 0.28; [138] 2.14 ± 1.67) as compared to script-driven imagery studies inducing dissociation (e.g., [55]: BPDd 6.85 ± 2.03; 9-point Likert scale, 9 indicating extreme symptoms). More research is needed to examine whether effects of dissociation on “affective brain regions” (e.g., amygdala) may become particularly evident in highly stressful situations and during high state dissociation in BPD. This may have implications for neurobiological conceptualizations of the disorder, as amygdala hyperreactivity to emotional stimuli is assumed to be a key feature of BPD [110•, 111•]. Assuming that dissociation dampens amygdala reactivity to emotional stimuli, the presence of dissociative symptoms may partly (aside from other factors such as medication status [111•]) explain why some previous studies did not replicate amygdala hyperreactivity in BPD [110•].

Apart from the amygdala, the left IFG, which has been implicated in interference inhibition and suppression of impulses [104], may be implicated in dissociative states in BPD. Three script-driven imagery studies consistently found increased activity in this area in patients listening to a dissociation script [59] and performing affective–cognitive tasks after dissociation induction [55, 60•]. As no clinical control groups were included in these studies and a high percentage of patients [59, 60•] or all patients [55] respectively reported a history of trauma, it remains unclear whether these findings are specific to BPD. Increased activity in the IFG [10] and stronger IFG FC with right cingulate gyrus [131] was also observed in D-PTSD exposed to autobiographical trauma scripts [57].

In addition, there is primary evidence for an association between trait dissociation and alterations in regions of the DMN (subsystems) such as the PCC, precuneus, hippocampus, and dorsal PFC in BPD. As previously mentioned, the DMN has been associated with “inward-directed” (self-referential) processes [79–81]. Again, it remains unclear whether these alterations in FC are specific to BPD or rather a trans-diagnostic phenomenon, as alterations in DMN (regions) were also found in depersonalization disorder [78•] and D-PTSD [87•].

## Overall Discussion and Conclusion

In this article, we aimed to (1) provide an overview of neurobiological models of dissociation and neuroimaging research in depersonalization disorder, DID, and D-PTSD and (2) to give an overview of recent neuroimaging studies in BPD that examined links between dissociation and altered brain function/structure.

Pathological dissociation is a complex and heterogeneous phenomenon [2–4, 153], which has been closely linked to traumatic stress [1, 10]. Dissociation may be a protective strategy to cope with overwhelming emotions in traumatic/stressful situations [1, 3, 10]. The cost of this subjective detachment appears to be a disruption of mental functions that are crucial to the development of identity, self-control, and emotion regulation [1, 16, 36, 37, 116].

The precise neurobiological underpinnings of dissociation remain elusive, but there is evidence for a link between dissociative states/traits and altered (co)activity in brain regions involved in emotion processing and memory (e.g., amygdala, hippocampus, parahippocampal gyrus, and middle/superior temporal gyrus), interoception and attention regulation (insula), self-referential processes (e.g., PCC and precuneus), cognitive control, and arousal modulation (e.g., mPFC, IFC, and ACC)—functions which may be altered during dissociation [1, 9, 10, 11•].

Future studies may address how changes in brain activity during dissociation are related to altered neuropsychological/cognitive functioning, e.g., encoding and subsequent recall of emotional information [110•, 111•]. It has been proposed that dissociation is associated with diminished recollection of trauma-related emotional information, although heterogeneous findings were reported [154–156]. The combination of dissociation induction and affective–cognitive neuropsychological tasks in neuroimaging research may contribute to a better understanding of this relationship.

As pointed out before, interpretations of the abovementioned research are complicated by methodological aspects (e.g., comorbidities, shared etiological factors, psychotropic medication, and differences in gender distribution) but also by conceptual differences: Dissociation involves a broad range of psychological and somatoform symptoms [2–4, 153]. A more precise differentiation between dissociative symptoms in future neuroimaging research may help to identify more specific associations [44•].

Most studies, summarized in this article, used well-established psychometric scales of dissociation (e.g., DES or DSS), showing excellent internal consistency, reliability, and high specificity and sensitivity to change in symptomatology [53]. As mentioned above, correlations (e.g., between neuroimaging findings and scores on these scales) give an estimate of the strength of a relationship (e.g., between brain structure/function and dissociative symptoms) but do not allow causal conclusions: it remains unclear whether alterations are a predisposition for or the result of frequent dissociation and probably stem from an interplay of multiple factors. Longitudinal studies and/or studies applying approaches like dynamic causal modeling are needed to gain more insight into possible causal relationships. Moreover, other variables that were not assessed in these studies might have moderated (strengthened/weakened) or mediated (explained) the relationship. To further clarify this, additional longitudinal studies, addressing possible moderating or mediating factors (e.g., trauma), are needed. Further limitations include small sample sizes and sole inclusion of female samples. To gain clearer insight into the role of dissociation in brain structure and function, studies with larger data sets in both female and male patients and meta-analyses are needed to replicate/extend existing findings.

### Possible Clinical Implications

Neuroimaging research on dissociation in BPD can have important clinical implications. Dissociative symptoms were found to hinder treatment outcome, possibly by interfering with habituation processes and new learning: such a negative effect of dissociative symptoms on treatment outcome has been shown for several disorders [46–50, 129•]. While dissociation can be adaptive in certain situations (e.g., in threatening situations that cannot be escaped or controlled), generalized and involuntary dissociative responses can have detrimental effects across multiple life domains [9]. Tailored interventions may help individuals to reduce/control dissociation at moments when such processes are disruptive and therefore maladaptive.

So far, neuroimaging studies have mainly focused on identifying the neural processes possibly underlying dissociation. As a next step, neuroimaging research may help to identify neural changes associated with a dissociative-symptom reduction after tailored interventions [148] and psychotherapy outcome [157–159].

Neuroimaging tools such as real-time fMRI may be used to translate knowledge on possible neural pathways involved in dissociation into experimental interventions. Real-time fMRI neurofeedback might be a promising add-on tool in combination with pharmaco- and psychotherapeutic treatment [148]. As a first step, more pilot studies are needed to identify which brain regions or functional processes/signals may be suitable targets for such interventions, as so far very little is known about the neural mechanisms of change that are key modulators in this relationship.

## References

[1] Spiegel D, Loewenstein RJ, Lewis-Fernandez R, Sar V, Simeon D, Vermetten E (2011). Dissociative disorders in DSM-5. Depress Anxiety.

[2] Holmes EA, Brown RJ, Mansell W, Fearon RP, Hunter EC, Frasquilho F (2005). Are there two qualitatively distinct forms of dissociation? A review and some clinical implications. Clin Psychol Rev.

[3] Spiegel D, Cardena E (1991). Disintegrated experience: the dissociative disorders revisited. J Abnorm Psychol.

[4] Waller NG, Putnam FW, Bernstein CE (1996). Types of dissociation and dissociative types: a taxometric analysis of dissociative experiences. Psychol Methods.

[5] APA (2013). Diagnostic and statistical manual of mental disorders: DSM-5.

[6] Stiglmayr CE, Ebner-Priemer UW, Bretz J, Behm R, Mohse M, Lammers CH (2008). Dissociative symptoms are positively related to stress in borderline personality disorder. Acta Psychiatr Scand.

[7] Ebner-Priemer UW, Mauchnik J, Kleindienst N, Schmahl C, Peper M, Rosenthal MZ (2009). Emotional learning during dissociative states in borderline personality disorder. J Psychiatry Neuroci.

[8] Haaland VO, Landro NI (2009). Pathological dissociation and neuropsychological functioning in borderline personality disorder. Acta Psychiatr Scand.

[9] Lanius RA, Brand B, Vermetten E, Frewen PA, Spiegel D (2012). The dissociative subtype of posttraumatic stress disorder: rationale, clinical and neurobiological evidence, and implications. Depress Anxiety.

[10] Lanius RA, Vermetten E, Loewenstein RJ, Brand B, Schmahl C, Bremner JD (2010). Emotion modulation in PTSD: clinical and neurobiological evidence for a dissociative subtype. Am J Psychiatr.

[11] Vermetten E, Spiegel D (2014). Trauma and dissociation: implications for borderline personality disorder. Curr Psychiatry Rep.

[12] Elbert T, Rockstroh B, Kolassa IT, Schauer M, Neuner F, Baltes P, Reuter-Lorenz P, Rösler F (2006). The influence of organized violence and terror on brain and mind—a co-constructive perspective. Lifespan development and the brain: the perspective of biocultural co-constructivism.

[13] Ford JD, Courtois CA (2014). Complex PTSD, affect dysregulation, and borderline personality disorder. Borderline Personal Disord Emot Dysregul.

[14] Gershuny BS, Thayer JF (1999). Relations among psychological trauma, dissociative phenomena, and trauma-related distress: a review and integration. Clin Psychol Rev.

[15] Nijenhuis ERS, Spinhoven P, van Dyck R, van der Hart O, Vanderlinden J (1998). Degree of somatoform and psychological dissociation in dissociative disorder is correlated with reported trauma. J Trauma Stress.

[16] Schauer M, Elbert T (2010). Dissociation following traumatic stress: etiology and treatment. J Psychol.

[17] van der Kolk BA, van der Hart O (1989). Pierre Janet and the breakdown of adaptation in psychological trauma. Am J Psychiatr.

[18] Marmar CR, Weiss DS, Metzler TJ, Bremner JD, Marmar CR (1998). Peritraumatic dissociation and posttraumatic stress disorder. Trauma, memory and dissociation.

[19] Pieper S, Out D, Bakermans-Kranenburg MJ, van Ijzendoorn MH (2011). Behavioral and molecular genetics of dissociation: the role of the serotonin transporter gene promoter polymorphism (5-HTTLPR). J Trauma Stress.

[20] Wolf EJ, Rasmusson AM, Mitchell KS, Logue MW, Baldwin CT, Miller MW (2014). A genome-wide association study of clinical symptoms of dissociation in a trauma-exposed sample. Depress Anxiety.

[21] Hagenaars MA, Oitzl M, Roelofs K (2014). Updating freeze: aligning animal and human research. Neurosci Biobehav Rev.

[22] Cannon WB (1929). Bodily changes in pain, hunger, fear, and range.

[23] Fanselow MS, Lester LS, Bolles RC, Breecher MD (1988). A functional behavioristic approach to aversively modtivated behavior: predatory immenence as a determinant of the topography of the defensive behavior. Evolution and learning.

[24] Nesse RM (1999). Proximate and evolutionary studies of anxiety, stress and depression: synergy at the interface. Neurosci Biobehav Rev.

[25] Bovin MJ, Marx BP (2011). The importance of the peritraumatic experience in defining traumatic stress. Psychol Bull.

[26] Candel I, Merckelbach H (2004). Peritraumatic dissociation as a predictor of post-traumatic stress disorder: a critical review. Compr Psychiatry.

[27] Lensvelt-Mulders G, van der Hart O, van Ochten JM, van Son MJ, Steele K, Breeman L (2008). Relations among peritraumatic dissociation and posttraumatic stress: a meta-analysis. Clin Psychol Rev.

[28] van der Hart O, van Ochten JM, van Son MJ, Steele K, Lensvelt-Mulders G (2008). Relations among peritraumatic dissociation and posttraumatic stress: a critical review. J Trauma Dissociation.

[29] Van der Kolk BA, McFarlane AC, Weisaeth L (1996). Traumatic stress: the effects of overwhelming experience on mind, body, and society.

[30] van der Velden PG, Wittmann L (2008). The independent predictive value of peritraumatic dissociation for PTSD symptomatology after type I trauma: a systematic review of prospective studies. Clin Psychol Rev.

[31] Bremner JD (2006). Traumatic stress: effects on the brain. Dialogues Clin Neurosci.

[32] Bremner JD, Williams LM, Banyard V (1999). Traumatic memories lost and found: can lost memory of abuse be found in the brain?. Trauma & memory.

[33] Elzinga BM, Bremner JD (2002). Are the neural substrates of memory the final common pathway in posttraumatic stress disorder (PTSD)?. J Affect Disord.

[34] Krystal JH, Bennett A, Bremner JD, Southwick SM, Charney DS, Michelson LK, Ray WJ (1996). Recent developments in the neurobiology of dissociation. Implications for posttraumatic stress disorder. Handbook of dissociation theoretical, empirical, and clinical perspectives.

[35] Conway MA, Pleydell-Pearce CW (2000). The construction of autobiographical memories in the self-memory system. Psychol Rev.

[36] Petersen SE, Posner MI (2012). The attention system of the human brain: 20 years after. Annu Rev Neurosci.

[37] Rueda MR, Posner MI, Rothbart MK (2005). The development of executive attention: contributions to the emergence of self-regulation. Dev Neuropsychol.

[38] Bremner JD, Vermetten E, Southwick SM, Krystal JH, Charney DS. Trauma, memory, and dissociation: an integrative formulation. American Psychiatric Association Press. 1998:365–402.

[39] Brewin CR (2001). A cognitive neuroscience account of posttraumatic stress disorder and its treatment. Behav Res Ther.

[40] Brewin CR, Dalgleish T, Joseph S (1996). A dual representation theory of posttraumatic stress disorder. Psychol Rev.

[41] Ehlers A, Clark DM (2000). A cognitive model of posttraumatic stress disorder. Behav Res Ther.

[42] Foa EB, Riggs DS (1995). Posttraumatic-stress-disorder following assault—theoretical considerations and empirical-findings. Curr Dir Psychol Sci.

[43] Frewen PA, Lanius RA (2006). Neurobiology of dissociation: unity and disunity in mind-body-brain. Psychiatr Clin North Am.

[44] Frewen PA, Lanius RA (2014). Trauma-related altered states of consciousness: exploring the 4-D model. J Trauma Dissociation.

[45] Bennett DC, Modrowski CA, Kerig PK, Chaplo SD (2015). Investigating the dissociative subtype of posttraumatic stress disorder in a sample of traumatized detained youth. Psychol Trauma.

[46] Michelson L, Vives A, Testa S, Marchione N, June K (1998). The role of trauma and dissociation in cognitive-behavioral psychotherapy outcome and maintenance for panic disorder with agoraphobia. Behav Res Ther.

[47] Rufer M, Held D, Cremer J, Fricke S, Moritz S, Peter H (2006). Dissociation as a predictor of cognitive behavior therapy outcome in patients with obsessive-compulsive disorder. Psychother Psychosom.

[48] Spitzer C, Barnow S, Freyberger HJ, Grabe HJ (2007). Dissociation predicts symptom-related treatment outcome in short-term inpatient psychotherapy. Aust N Z J Psychiatry.

[49] Kleindienst N, Limberger MF, Ebner-Priemer UW, Keibel-Mauchnik J, Dyer A, Berger M (2011). Dissociation predicts poor response to dialectial behavioral therapy in female patients with borderline personality disorder. J Personal Disord.

[50] Spitzer C, Barnow S, Freyberger HJ, Grabe HJ (2007). Dissociation predicts symptom-related treatment outcome in short-term inpatient psychotherapy. Aust N Z J Psychiatry.

[51] Bernstein EM, Putnam FW (1986). Development, reliability, and validity of a dissociation scale. J Nerv Ment Dis.

[52] Stiglmayr CE, Braakmann D, Haaf B, Stieglitz RD, Bohus M (2003). Development and characteristics of dissociation-tension-scale acute (DSS-Akute). Psychother Psychosom Med Psychol.

[53] Stiglmayr C, Schimke P, Wagner T, Braakmann D, Schweiger U, Sipos V (2010). Development and psychometric characteristics of the dissociation tension scale. J Pers Assess.

[54] Stiglmayr C, Schmahl C, Bremner JD, Bohus M, Ebner-Priemer U (2009). Development and psychometric characteristics of the DSS-4 as a short instrument to assess dissociative experience during neuropsychological experiments. Psychopathology.

[55] Krause-Utz A, Winter D, Schriner F, Chiu C-D, Lis S, Spinhoven P, et al. Reduced amygdala activity and emotional distractibility during dissociation in borderline personality disorder. Submitted.

[56] Lanius RA, Williamson PC, Bluhm RL, Densmore M, Boksman K, Neufeld RW (2005). Functional connectivity of dissociative responses in posttraumatic stress disorder: a functional magnetic resonance imaging investigation. Biol Psychiatry.

[57] Lanius RA, Williamson PC, Boksman K, Densmore M, Gupta M, Neufeld RW (2002). Brain activation during script-driven imagery induced dissociative responses in PTSD: a functional magnetic resonance imaging investigation. Biol Psychiatry.

[58] Lanius RA, Williamson PC, Densmore M, Boksman K, Neufeld RW, Gati JS (2004). The nature of traumatic memories: a 4-T FMRI functional connectivity analysis. Am J Psychiatr.

[59] Ludascher P, Valerius G, Stiglmayr C, Mauchnik J, Lanius RA, Bohus M (2010). Pain sensitivity and neural processing during dissociative states in patients with borderline personality disorder with and without comorbid posttraumatic stress disorder: a pilot study. J Psychiatry Neurosci : JPN.

[60] • Winter D, Krause-Utz A, Lis S, Chiu CD, Lanius RA, Schriner F, et al. Dissociation in borderline personality disorder: Disturbed cognitive and emotional inhibition and its neural correlates.Psychiatry Res. 2015;233(3):339–51. **Script-driven fMRI study on the impact of dissociation on cognitive control in BPD**. 10.1016/j.pscychresns.2015.05.018 26254542

[61] Krystal JH, Bremner JD, SouthwickSM, Charney DS. The emerging neurobiology of dissociation: implications for treatment of posttraumatic stress disorder. In: Bremner JD, Marmar CR, editors. 1. Washington DC: American Psychiatric Press; 1998. p. 321–63.

[62] Sierra M, Berrios GE. Depersonalization: neurobiological perspectives. Biol Psychiatry. 1998;44(9):898–908. 10.1016/s0006-3223(98)00015-8 9807645

[63] Ochsner KN, Gross JJ. The neural architecture of emotion regulation. In: Gross JJ, Buck R, editors. The Handbook of Emotion Regulation. New York: Guilford Press; 2007. p. 87–109.

[64] Phillips ML, Drevets WC, Rauch SL, Lane R. Neurobiology of emotion perception I: The neural basis of normal emotion perception. Biol Psychiatry. 2003;54(5):504–14. 10.1016/s0006-3223(03)00168-9 12946879

[65] Sierra M, Senior C, Dalton J, McDonough M, Bond A, Phillips ML (2002). Autonomic response in depersonalization disorder. Arch Gen Psychiatry.

[66] Sierra M, Senior C, Phillips ML, David AS (2006). Autonomic response in the perception of disgust and happiness in depersonalization disorder. Psychiatry Res.

[67] Davis M, Whalen PJ (2001). The amygdala: Vigilance and emotion. Mol Psychiatry.

[68] Phan KL, Wager TD, Taylor SF, Liberzon I (2004). Functional neuroimaging studies of human emotions. CNS Spectr.

[69] Phillips ML, Medford N, Senior C, Bullmore ET, Suckling J, Brammer MJ (2001). Depersonalization disorder: thinking without feeling. Psychiatry Res.

[70] Phillips ML, Sierra M (2003). Depersonalization disorder: a functional neuroanatomical perspective. Stress.

[71] Critchley HD, Mathias CJ, Dolan RJ (2001). Neural activity in the human brain relating to uncertainty and arousal during anticipation. Neuron.

[72] Damasio AR, Grabowski TJ, Bechara A, Damasio H, Ponto LLB, Parvizi J (2000). Subcortical and cortical brain activity during the feeling of self-generated emotions. Nat Neurosci.

[73] Dosenbach NU, Visscher KM, Palmer ED, Miezin FM, Wenger KK, Kang HC (2006). A core system for the implementation of task sets. Neuron.

[74] Menon V (2011). Large-scale brain networks and psychopathology: a unifying triple network model. Trends Cogn Sci.

[75] Menon V, Uddin LQ (2010). Saliency, switching, attention and control: a network model of insula function. Brain Struct Funct.

[76] Sedeno L, Couto B, Melloni M, Canales-Johnson A, Yoris A, Baez S (2014). How do you feel when you can’t feel your body? Interoception, functional connectivity and emotional processing in depersonalization-derealization disorder. PLoS One.

[77] Lemche E, Brammer MJ, David AS, Surguladze SA, Phillips ML, Sierra M (2013). Interoceptive-reflective regions differentiate alexithymia traits in depersonalization disorder. Psychiatry Res.

[78] Lemche E, Sierra-Siegert M, David AS, Phillips ML, Gasston D, Williams SC (2016). Cognitive load and autonomic response patterns under negative priming demand in depersonalization-derealization disorder. Eur J Neurosci.

[79] Greicius M (2008). Resting-state functional connectivity in neuropsychiatric disorders. Curr Opin Neurol.

[80] Greicius MD, Krasnow B, Reiss AL, Menon V (2003). Functional connectivity in the resting brain: a network analysis of the default mode hypothesis. Proc Natl Acad Sci USA.

[81] Raichle ME, MacLeod AM, Snyder AZ, Powers WJ, Gusnard DA, Shulman GL (2001). A default mode of brain function. Proc Natl Acad Sci USA.

[82] Ketay S, Hamilton HK, Haas BW, Simeon D (2014). Face processing in depersonalization: an fMRI study of the unfamiliar self. Psychiatry Res.

[83] Simeon D, Guralnik O, Hazlett EA, Spiegel-Cohen J, Hollander E, Buchsbaum MS (2000). Feeling unreal: a PET study of depersonalization disorder. Am J Psychiatr.

[84] Spiegel D (1991). Neurophysiological correlates of hypnosis and dissociation. J Neuropsychiatry Clin Neurosci.

[85] Krystal JH, Bennett AL, Bremner JD, Southwick SM, Charney DS, Friedman MJ, Charney DS (1995). Toward a cognitive neuroscience of dissociation and altered memory functions in post-traumatic stress disorder. Deutch AY, editors.

[86] Felmingham K, Kemp AH, Williams L, Falconer E, Olivieri G, Peduto A (2008). Dissociative responses to conscious and non-conscious fear impact underlying brain function in post-traumatic stress disorder. Psychol Med.

[87] Tursich M, Ros T, Frewen PA, Kluetsch RC, Calhoun VD, Lanius RA (2015). Distinct intrinsic network connectivity patterns of post-traumatic stress disorder symptom clusters. Acta Psychiatr Scand.

[88] Sridharan D, Levitin DJ, Menon V (2008). A critical role for the right fronto-insular cortex in switching between central-executive and default-mode networks. Proc Natl Acad Sci USA.

[89] Nicholson AA, Densmore M, Frewen PA, Theberge J, Neufeld RW, McKinnon MC (2015). The dissociative subtype of posttraumatic stress disorder: unique resting-state functional connectivity of basolateral and centromedial amygdala complexes. Neuropsychopharmacology.

[90] Reinders AA, Nijenhuis ER, Quak J, Korf J, Haaksma J, Paans AM (2006). Psychobiological characteristics of dissociative identity disorder: a symptom provocation study. Biol Psychiatry.

[91] Reinders AA, Willemsen AT, den Boer JA, Vos HP, Veltman DJ, Loewenstein RJ (2014). Opposite brain emotion-regulation patterns in identity states of dissociative identity disorder: a PET study and neurobiological model. Psychiatry Res.

[92] Schlumpf YR, Reinders AA, Nijenhuis ER, Luechinger R, van Osch MJ, Jancke L (2014). Dissociative part-dependent resting-state activity in dissociative identity disorder: a controlled FMRI perfusion study. PLoS One.

[93] Daniels JK, Gaebler M, Lamke JP, Walter H (2015). Grey matter alterations in patients with depersonalization disorder: a voxel-based morphometry study. J Psychiatry Neurosci.

[94] Vermetten E, Schmahl C, Lindner S, Loewenstein RJ, Bremner JD (2006). Hippocampal and amygdalar volumes in dissociative identity disorder. Am J Psychiatr.

[95] Ehling T, Nijenhuis ER, Krikke AP (2008). Volume of discrete brain structures in complex dissociative disorders: preliminary findings. Prog Brain Res.

[96] Weniger G, Lange C, Sachsse U, Irle E (2008). Amygdala and hippocampal volumes and cognition in adult survivors of childhood abuse with dissociative disorders. Acta Psychiatr Scand.

[97] Dannlowski U, Stuhrmann A, Beutelmann V, Zwanzger P, Lenzen T, Grotegerd D (2012). Limbic scars: long-term consequences of childhood maltreatment revealed by functional and structural magnetic resonance imaging. Biol Psychiatry.

[98] Daniels JK, Frewen P, Theberge J, Lanius RA (2016). Structural brain aberrations associated with the dissociative subtype of post-traumatic stress disorder. Acta Psychiatr Scand.

[99] Karl A, Schaefer M, Malta LS, Dorfel D, Rohleder N, Werner A (2006). A meta-analysis of structural brain abnormalities in PTSD. Neurosci Biobehav Rev.

[100] Woon FL, Hedges DW (2009). Amygdala volume in adults with posttraumatic stress disorder: a meta-analysis. J Neuropsychiatry Clin Neurosci.

[101] Nardo D, Hogberg G, Lanius RA, Jacobsson H, Jonsson C, Hallstrom T (2013). Gray matter volume alterations related to trait dissociation in PTSD and traumatized controls. Acta Psychiatr Scand.

[102] • Basmaci Kandemir S, Bayazit H, Selek S, Kilicaslan N, Kandemir H, Karababa IF, et al. Tracking down the footprints of bad paternal relationships in dissociative disorders: a diffusion tensor imaging study. J Trauma Dissociation. 2015:1–11. **Study investigating white matter alterations in dissociative disorders**. 10.1080/15299732.2015.1111282 26566870

[103] Chalavi S, Vissia EM, Giesen ME, Nijenhuis ER, Draijer N, Barker GJ (2015). Similar cortical but not subcortical gray matter abnormalities in women with posttraumatic stress disorder with versus without dissociative identity disorder. Psychiatry Res.

[104] Aron AR, Robbins TW, Poldrack RA (2014). Inhibition and the right inferior frontal cortex: one decade on. Trends Cogn Sci.

[105] Goldman-Rakic PS, Bates JF, Chafee MV (1992). The prefrontal cortex and internally generated motor acts. Curr Opin Neurobiol.

[106] Krause-Utz A, Schmahl C (2016). A more global look at altered neural structure and resting-state function in borderline personality disorder. Biol Psychiatry.

[107] Korzekwa MI, Dell PF, Links PS, Thabane L, Fougere P (2009). Dissociation in borderline personality disorder: a detailed look. J Trauma Dissociation: Off J Int Soc Study Dissociation.

[108] APA. Diagnostic and statistical manual of mental disorders, fourth edition, Text Revision (DSM-IV-TR). Washington, DC: American Psychiatric Association; 2000.

[109] Carpenter RW, Trull TJ (2013). Components of emotion dysregulation in borderline personality disorder: a review. Curr Psychiatry Rep.

[110] Krause-Utz A, Winter D, Niedtfeld I, Schmahl C (2014). The latest neuroimaging findings in borderline personality disorder. Curr Psychiatry Rep.

[111.•] Schulze L, Schmahl C, Niedtfeld I (2016). Neural correlates of disturbed emotion processing in borderline personality disorder: a multimodal meta-analysis. Biol Psychiatry.

[112] van Zutphen L, Siep N, Jacob GA, Goebel R, Arntz A (2015). Emotional sensitivity, emotion regulation and impulsivity in borderline personality disorder: a critical review of fMRI studies. Neurosci Biobehav Rev.

[113] Chopra HD, Beatson JA (1986). Psychotic symptoms in borderline personality disorder. Am J Psychiatr.

[114] Skodol AE, Gunderson JG, Pfohl B, Widiger TA, Livesley WJ, Siever LJ (2002). The borderline diagnosis I: psychopathology comorbidity, and personality structure. Biol Psychiatry.

[115] Korzekwa MI, Dell PF, Pain C (2009). Dissociation and borderline personality disorder: an update for clinicians. Current Psychiatry Reports.

[116] Simeon D, Nelson D, Elias R, Greenberg J, Hollander E (2003). Relationship of personality to dissociation and childhood trauma in borderline personality disorder. CNS Spectrums.

[117] Zanarini MC, Frankenburg FR, Jager-Hyman S, Reich DB, Fitzmaurice G (2008). The course of dissociation for patients with borderline personality disorder and axis II comparison subjects: a 10-year follow-up study. Acta Psychiatr Scand.

[118] Zanarini MC, Ruser TF, Frankenburg FR, Hennen J, Gunderson JG (2000). Risk factors associated with the dissociative experiences of borderline patients. J Nerv Ment Dis.

[119] Banich MT, Mackiewicz KL, Depue BE, Whitmer AJ, Miller GA, Heller W (2009). Cognitive control mechanisms, emotion and memory: a neural perspective with implications for psychopathology. Neurosci Biobehav Rev.

[120] Ludascher P, Bohus M, Lieb K, Philipsen A, Jochims A, Schmahl C (2007). Elevated pain thresholds correlate with dissociation and aversive arousal in patients with borderline personality disorder. Psychiatry Res.

[121] Dutra L, Bureau JF, Holmes B, Lyubchik A, Lyons-Ruth K (2009). Quality of early care and childhood trauma: a prospective study of developmental pathways to dissociation. J Nerv Ment Dis.

[122] Ross-Gower J, Waller G, Tyson M, Elliott P (1998). Reported sexual abuse and subsequent psychopathology among women attending psychology clinics: the mediating role of dissociation. Br J Clin Psychol.

[123] Shearer SL (1994). Dissociative phenomena in women with borderline personality disorder. Am J Psychiatr.

[124] Van Den Bosch LM, Verheul R, Langeland W, Van Den Brink W (2003). Trauma, dissociation, and posttraumatic stress disorder in female borderline patients with and without substance abuse problems. Aust N Z J Psychiatry.

[125] Dalenberg CJ, Glaser D, Alhassoon OM (2012). Statistical support for subtypes in posttraumatic stress disorder: the how and why of subtype analysis. Depress Anxiety.

[126] Ogawa JR, Sroufe LA, Weinfield NS, Carlson EA, Egeland B (1997). Development and the fragmented self: longitudinal study of dissociative symptomatology in a nonclinical sample. Dev Psychopathol.

[127] Brand BL, Lanius RA (2014). Chronic complex dissociative disorders and borderline personality disorder: disorders of emotion dysregulation?. Borderline Personal Disord Emot Dysregul.

[128] Arntz A, Stupar-Rutenfrans S, Bloo J, van Dyck R, Spinhoven P (2015). Prediction of treatment discontinuation and recovery from borderline personality disorder: results from an RCT comparing schema therapy and transference focused psychotherapy. Behav Res Ther.

[129] Winter D, Elzinga B, Schmahl C (2014). Emotions and memory in borderline personality disorder. Psychopathology.

[130] • Krause-Utz A, Veer IM, Rombouts SARB, Bohus M, Schmahl C, Elzinga BM. Amygdala and anterior cingulate resting-state functional connectivity in borderline personality disorder patients with a history of interpersonal trauma. Psychological Medicine. 2014:1–13. **FMRI study investigating links between dissociation and resting-state functional connectivity in BPD**. 10.1017/S0033291714000324 25066544

[131] Lange C, Kracht L, Herholz K, Sachsse U, Irle E (2005). Reduced glucose metabolism in temporo-parietal cortices of women with borderline personality disorder. Psychiatry Res.

[132] Sar V, Unal SN, Ozturk E (2007). Frontal and occipital perfusion changes in dissociative identity disorder. Psychiatry Res.

[133] Wolf RC, Thomann PA, Sambataro F, Vasic N, Schmid M, Wolf ND (2012). Orbitofrontal cortex and impulsivity in borderline personality disorder: an MRI study of baseline brain perfusion. Eur Arch Psychiatry Clin Neurosci.

[134] Wolf RC, Sambataro F, Vasic N, Schmid M, Thomann PA, Bienentreu SD (2011). Aberrant connectivity of resting-state networks in borderline personality disorder. J Psychiatr Neurosci: JPN.

[135] Hazlett EA, Zhang J, New AS, Zelmanova Y, Goldstein KE, Haznedar MM (2012). Potentiated amygdala response to repeated emotional pictures in borderline personality disorder. Biol Psychiatry.

[136] Kluetsch RC, Schmahl C, Niedtfeld I, Densmore M, Calhoun VD, Daniels J (2012). Alterations in default mode network connectivity during pain processing in borderline personality disorder. Arch Gen Psychiatry.

[137] Kraus A, Esposito F, Seifritz E, Di Salle F, Ruf M, Valerius G (2009). Amygdala deactivation as a neural correlate of pain processing in patients with borderline personality disorder and co-occurrent posttraumatic stress disorder. Biol Psychiatry.

[138] Krause-Utz A, Keibel-Mauchnik J, Ebner-Priemer U, Bohus M, Schmahl C. Classical conditioning in borderline personality disorder: an fMRI study. Eur Arch Psychiatry Clin Neurosci. 2015. 10.1007/s00406-015-0593-1 25814470

[139] Krause-Utz A, Oei NY, Niedtfeld I, Bohus M, Spinhoven P, Schmahl C (2012). Influence of emotional distraction on working memory performance in borderline personality disorder. Psychol Med.

[140] Wingenfeld K, Rullkoetter N, Mensebach C, Beblo T, Mertens M, Kreisel S (2009). Neural correlates of the individual emotional Stroop in borderline personality disorder. Psychoneuroendocrinology.

[141] • Paret C, Kluetsch R, Zaehringer J, Ruf M, Demirakca T, Bohus M, et al. Alterations of amygdala-prefrontal connectivity with real-time fMRI neurofeedback in BPD patients. Soc Cogn Affect Neurosci. 2016. **Real-time fMRI study investigating the effect of neurofeedback on amygdala activity and functional connectivity in BPD**. 10.1093/scan/nsw016 PMC4884315 26833918

[142] Patton JH, Stanford MS, Barratt ES. Factor structure of the Barratt impulsiveness scale. J Clin Psychol. 1995;51(6):768–74 10.1002/1097-4679(199511)51:6<768::aid-jclp2270510607>3.0.co;2-1 8778124

[143] Hoerst M, Weber-Fahr W, Tunc-Skarka N, Ruf M, Bohus M, Schmahl C (2010). Correlation of glutamate levels in the anterior cingulate cortex with self-reported impulsivity in patients with borderline personality disorder and healthy controls. Arch Gen Psychiatry.

[144] Ende G, Cackowski S, Van Eijk J, Sack M, Demirakca T, Kleindienst N (2016). Impulsivity and aggression in female BPD and ADHD patients: association with ACC glutamate and GABA concentrations. Neuropsychopharmacology.

[145] Krause-Utz A, Elzinga BM, Oei NY, Paret C, Niedtfeld I, Spinhoven P (2014). Amygdala and dorsal anterior cingulate connectivity during an emotional working memory task in borderline personality disorder patients with interpersonal trauma history. Front Hum Neurosci.

[146] Friston KJ, Buechel C, Fink GR, Morris J, Rolls E, Dolan RJ (1997). Psychophysiological and modulatory interactions in neuroimaging. Neuroimage.

[147] Gratz KL, Roemer L (2004). Multidimensional assessment of emotion regulation and dysregulation: development, factor structure, and initial validation of the difficulties in emotion regulation scale. J Psychopathol Behav Assess 26:41–54. doi:10.1007/s10862-008-9102-4

[148] Lanius RA (2015). Trauma-related dissociation and altered states of consciousness: a call for clinical, treatment, and neuroscience research. Eur J Psychotraumatol.

[149] Irle E, Lange C, Sachsse U (2005). Reduced size and abnormal asymmetry of parietal cortex in women with borderline personality disorder. Biol Psychiatry.

[150] • Niedtfeld I, Schulze L, Krause-Utz A, Demirakca T, Bohus M, Schmahl C. Voxel-based morphometry in women with borderline personality disorder with and without comorbid posttraumatic stress disorder. PLoS One. 2013;8(6):e65824. **Study investigating links between dissociation and brain structure in BPD**. 10.1371/journal.pone.0065824 PMC3680473 23776553

[151] Rusch N, Weber M, Il’yasov KA, Lieb K, Ebert D, Hennig J (2007). Inferior frontal white matter microstructure and patterns of psychopathology in women with borderline personality disorder and comorbid attention-deficit hyperactivity disorder. NeuroImage.

[152] Steinberg M (1994). Interviewers guide to the structured clinical interview for DSM-IV dissociative disorders.

[153] van der Hart O, Nijenhuis E, Steele K, Brown D (2004). Trauma-related dissociation: conceptual clarity lost and found. Aust N Z J Psychiatry.

[154] Elzinga BM, Ardon AM, Heijnis MK, De Ruiter MB, Van Dyck R, Veltman DJ (2007). Neural correlates of enhanced working-memory performance in dissociative disorder: a functional MRI study. Psychol Med.

[155] de Ruiter MB, Phaf RH, Elzinga BM, van Dyck R (2004). Dissociative style and individual differences in verbal working memory span. Conscious Cogn.

[156] Chiu CD, Yeh YY, Huang YM, Wu YC, Chiu YC (2009). The set switching function of nonclinical dissociators under negative emotion. J Abnorm Psychol.

[157] Schnell K, Herpertz SC (2007). Effects of dialectic-behavioral-therapy on the neural correlates of affective hyperarousal in borderline personality disorder. J Psychiatr Res.

[158] Goodman M, Carpenter D, Tang CY, Goldstein KE, Avedon J, Fernandez N (2014). Dialectical behavior therapy alters emotion regulation and amygdala activity in patients with borderline personality disorder. J Psychiatr Res.

[159] Winter D, Niedtfeld I, Schmitt R, Bohus M, Schmahl C, Herpertz SC. Neural correlates of distraction in borderline personality disorder before and after dialectical behavior therapy. Eur Arch Psychiatry Clin Neurosci. 2016 Apr 18. 10.1007/s00406-016-0689-2 27091455

